# Faunistic patterns of leaf beetles (Coleoptera, Chrysomelidae) within elevational and temporal gradients in Sierra de San Carlos, Mexico

**DOI:** 10.3897/zookeys.611.9608

**Published:** 2016-08-15

**Authors:** Uriel Jeshua Sánchez-Reyes, Santiago Niño-Maldonado, Ludivina Barrientos-Lozano, Shawn M. Clark, Robert W. Jones

**Affiliations:** 1 División de Estudios de Posgrado e Investigación. Instituto Tecnológico de Cd. Victoria. Boulevard Emilio Portes Gil No.1301, C.P. 87010. Ciudad Victoria, Tamaulipas, México; 2 Facultad de Ingeniería y Ciencias. Universidad Autónoma de Tamaulipas. Centro Universitario Victoria. CP. 87149. Victoria, Tamaulipas, México; 3 Brigham Young University, Monte L. Bean Life Science Museum, Provo, Utah 84602, U.S.A.; 4 Facultad de Ciencias Naturales. Universidad Autónoma de Querétaro. Avenida de las Ciencias, s/n, 76230 Juriquilla, Querétaro, México

**Keywords:** Biodiversity, chrysomelid beetles, ecological gradient, elevation, seasonality, sky island

## Abstract

The study of biodiversity of Chrysomelidae in Mexico and its variation within ecological gradients has increased recently, although important areas in the country remain to be explored. We conducted a faunistic inventory and analyzed the elevational and temporal variation of leaf beetle communities in the Sierra de San Carlos, in the state of Tamaulipas, in northeastern Mexico. This is an area with high to extreme priority for conservation, and due to its insular geographical position and to the vegetational communities present, it must be considered as a sky island. We selected seven sample sites distributed in different elevations within three localities, and comprising different vegetational communities. At each site, we randomly delimited 12 sample plots of 400 m^2^ where sampling was conducted by entomological sweep netting and collecting directly by hand. Sampling was conducted monthly at each plot, for a total of 1,008 samples between February 2013 and January 2014. By the end of the study, we had obtained a total of 3,081 specimens belonging to six subfamilies, 65 genera, and 113 species, with *Trichaltica scabricula* (Crotch, 1873) being recorded for first time in Mexico. Species richness was less than the values observed at other studies conducted in the same region, which is attributed to differences in the number of plant species and to the insular location of Sierra de San Carlos; however, the higher diversity values suggest a higher quality of natural resources and vegetational communities. No consistent pattern of leaf beetle communities was correlated with elevation, although higher values of species richness and diversity were obtained at the highest elevation site. The seasonal gradient showed that the rainy season is most favorable for leaf beetle communities. We found that species composition was different between sites and months, and also that there exists a significant association between the abundance obtained at each site and particular months. These results highlight the importance of different microhabitats for species distribution, and suggest that each species of Chrysomelidae has a differential response to environmental factors that vary within the elevational gradient and according to seasons. Also, we confirm and emphasize the important status of Sierra de San Carlos as a key natural area for biological conservation.

## Introduction

Chrysomelidae (excluding Bruchinae or seed beetles), whose members are also known as leaf beetles, is one of the most diverse taxa within Coleoptera, with more than 35,000 to 40,000 described species worldwide (Jolivet et al. 2009). As a predominantly phytophagous group, some species are important crop pests, while others are used efficiently to control weeds. This characteristic also makes them an important component of ecosystems, as they can compete with other herbivores (Gómez and González-Megías 2002). Also, leaf beetles have been used as indicators of regional biodiversity and environmental quality, and for monitoring changes in natural areas (Farrell and Erwin 1988, Flowers and Hanson 2003, Kalaichelvan and Verma 2005, Linzmeier et al. 2006, Baselga and Novoa 2007, Aslan and Ayvaz 2009). Because of their importance, numerous and detailed taxonomical works on Chrysomelidae had been conducted in North America north of Mexico, Central America, and South America (Riley et al. 2003). Recently, faunistic studies have been increasing for the Mexican leaf beetle fauna.

Mexico is located within an important geographic area, and the inhabiting fauna is the result of the interface of the Neotropical and Nearctic realms. So, the study of chrysomelid distribution in this region is useful to analyze the biogeographical and ecological patterns of its species in the American continent. In Mexico, the most explored and studied areas are the Baja California peninsula (Andrews and Gilbert 2005), the central and southern portions of the country, principally at the Biosphere Reserve of Sierra de Huautla (Ordóñez-Reséndiz and López-Pérez 2009, Ordóñez-Reséndiz et al. 2011, Ordóñez-Reséndiz et al. 2015), the state of Oaxaca (Furth 2013), and the state of Morelos (Burgos-Solorio and Anaya-Rosales 2004, Niño-Maldonado et al. 2016), where important faunistic and ecological data have been obtained. Other significant contributions have focused on the states of Jalisco (Niño-Maldonado et al. 2014b, Sandoval-Becerra et al. 2015), Hidalgo (Martínez-Sánchez et al. 2009, 2010), and Veracruz (Deloya and Ordóñez-Reséndiz 2008), and on the Sierra Tarahumara in Chihuahua (Furth 2009), as well as on country-wide studies of the tribe Alticini (Furth and Savini 1996, 1998, Furth 2006). To date, 2,174 species of Chrysomelidae are reported to be present in Mexico (Ordóñez-Reséndiz et al. 2014), but the increasing numbers of studies have provided new distribution data, as well as species recorded for the first time in the country (Medvedev et al. 2012, Moseyko et al. 2013, García-Robledo et al. 2014, López-Pérez et al. 2015, Sánchez-Reyes et al. 2015b). However, much of the faunistic information about the distribution and presence of the species in Mexico, including most recent compilations (i.e., Ordóñez-Reséndiz 2014, Niño-Maldonado et al. 2016), are based principally on documents and studies published at least over 30 years ago (Jacoby 1880, 1881, 1882, 1883, 1884, 1885, 1886, 1887, 1888a, 1888b, 1889, 1890, 1891, 1892, Baly 1885, 1886, Champion 1893, 1894, Blackwelder 1946, Wilcox 1975, Moldenke 1970), and from collection localities cited in original descriptions of species. Moreover, many of the species from these sources lack specific localities and were only labeled as “Mexico.” This clearly demonstrates the need for new faunistic studies which provide accurate data on the present distributions of chrysomelid species in Mexico.

Recently, a series of faunistic and ecological studies on leaf beetle fauna has been conducted in the northeastern portion of Mexico, specifically in the state of Tamaulipas. To date, 250 species have been recorded from this state, which now ranks fourth in chrysomelid diversity from Mexico (Niño-Maldonado et al. 2014a, Ordóñez-Reséndiz et al. 2014). Faunistic data from the state have been obtained from El Cielo Biosphere Reserve (Niño-Maldonado et al. 2005) and Peregrina Canyon, where detailed ecological and distributional patterns were also described (Sánchez-Reyes et al. 2014). Both areas are located within the Sierra Madre Oriental, and they are included in a protection category. Another very important area for biological conservation in Mexico and the northeastern portion of the country is the Sierra de San Carlos. This mountain range has been categorized as a terrestrial area with high to extreme priority for conservation, because of its insular geographical location within the northern gulf coastal plain and because of the relatively well-preserved nature of its natural resources (Arriaga et al. 2000, CONABIO 2007). Despite its biological interest, the only known studies from this area have focused on vegetation (Martínez 1998, Briones-Villarreal 1991), and only a few groups of insects (Meléndez-Jaramillo et al. 2014, 2015). Also, preliminary faunistic and ecological research on Chrysomelidae has been conducted there, showing interesting patterns and new species distributions (Sánchez-Reyes et al. 2015a).

Although data from faunistic inventories constitute a very important descriptor of diversity and allow analysis of species distribution from a region, it is also important that the variation of ecological patterns are associated with natural gradients, as they reflect the ecological and evolutionary adaptations of species to various environmental conditions (Ricklefs 2006, 2007). Elevation is one of the most studied gradients in species richness and diversity, because it dictates changes in environmental variables and abiotic factors such temperature, humidity, wind velocity, land area, and total radiation (Körner 2007, Sundqvist et al. 2013), which in turn determine distribution of species (Hodkinson 2005). The resulting patterns from these environmental influences are different according to the studied taxa, spatial scale, or geographic region, although evidence has shown that the most common pattern is a peak in diversity at mid-elevations (Lomolino 2001, Rahbek 2005, McCain and Grytnes 2010, Sanders and Rahbek 2012, Guo et al. 2013), including a peak in diversity of Chrysomelidae (Furth 2009, Sánchez-Reyes et al. 2014). Also, temporal and seasonal gradients, which determine various patterns of species diversity, are related to changes in elevation (Körner 2007). Indeed, factors that change according to elevation, such as temperature, humidity, and vegetation, are highly variable during seasonal and temporal succession in the same mountain (Barry 2008). All these characteristics show that mountains with their elevational gradients, such as Sierra de San Carlos, can be used as key natural scenarios for analysis of patterns of diversity and also for a future assessment of changes in distribution of leaf beetles and other taxa, related to climate change (McCain and Grytnes 2010, Sundqvist et al. 2013). Based on these criteria, the objectives of our study were to: 1) conduct a taxonomic inventory of chrysomelid species from the Sierra de San Carlos, Tamaulipas, Mexico; 2) analyze the elevational and seasonal patterns of species richness, abundance, and diversity of this taxon in the study area; and 3) identify the effects of the interaction between sites and months on leaf beetle communities.

## Methods

### Study area

**Figure 1.**
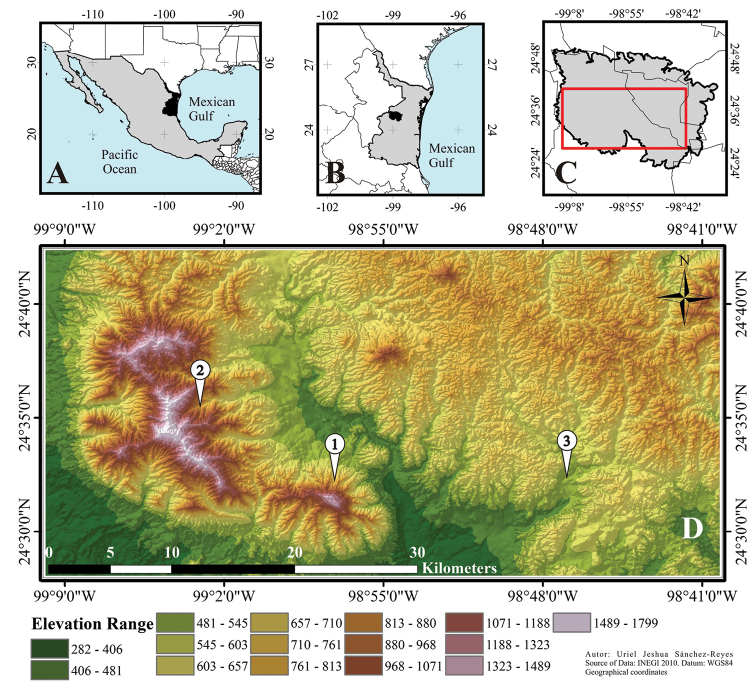
Study area. **A** Location of Tamaulipas in Mexico **B** Location of Sierra de San Carlos within Tamaulipas **C** Study area (red square) within Sierra de San Carlos **D** Details of study area: 1 = Cerro El Diente, 2 = Ejido Carricitos y Tinajas, 3 = San Nicolás.

The study was conducted in the Sierra de San Carlos (Figure 1), which includes the municipalities of Burgos, Cruillas, Jiménez, San Carlos, San Nicolás, and Villagran located in the central-west portion of the state of Tamaulipas, and also the municipality of Linares in the extreme eastern part of the state of Nuevo León, Mexico (Arriaga et al. 2000). The Sierra de San Carlos comprises an area of 2320 km^2^, being a polygon with the northwestern limit at 24°52.000'N, 99°12.067'W, and the southeastern limit at 24°23.050'N, 98°32.667'W. It constitutes an isolated mountain range within the Tamaulipas biogeographic province, bounded at the south by the Mexican gulf province (Morrone et al. 2002). Almost all vegetation types within the Sierra have a high conservation status, and they occur in principally temperate ecosystems (oak and pine forests) in the mountain areas, but there are also various types of tropical scrub vegetation in the lower areas, as well as other vegetational communities.

One of the most important characteristics of Sierra de San Carlos is its designation as a Priority Conservation Area in Mexico due to its biological, ecological and physiogeographical features: it is the northern limit of the Cloud Forest vegetation in Mexico, and it has some endemic plant species; also, it is considered as a biogeographical island (“sky island”) due to its isolation from other nearby mountain ranges, such as the Sierra Madre Oriental and Sierra de Tamaulipas (Arriaga et al. 2000). Accordingly, some areas with median, high and extreme conservation priority are located within Sierra de San Carlos (CONABIO 2007).

### Site location

We selected seven sampling sites distributed in three localities within Sierra de San Carlos (Figure 1D), including various elevations and vegetation types, as well as various conservation priorities. The first locality was Cerro El Diente which included four sampling sites: Site 1) containing submountain scrub vegetation, at a mean elevation of 550 masl; Site 2) consisting of Tamaulipan thorny scrub vegetation, at a mean elevation of 760 masl; Site 3) had oak forest vegetation, at a mean elevation of 960 masl; and Site 4) with Cloud Forest vegetation, at a mean elevation of 1080 masl. The second locality was Ejido Carricitos y Tinajas which had two sampling sites. These were: Site 5) with secondary elements of riparian vegetation, at a mean elevation of 730 masl; and Site 6) containing oak and pine forest vegetation, at a mean elevation of 820 masl. The third locality was San Nicolás with a single sampling site: Site 7) with submountain and Tamaulipan thorny scrub vegetation, at a mean elevation of 500 masl. Both the Cerro El Diente and Ejido Carricitos y Tinajas are located within areas with extreme conservation priority; the locality of San Nicolás belongs to a median conservation category (CONABIO 2007).

**Figure 2.**
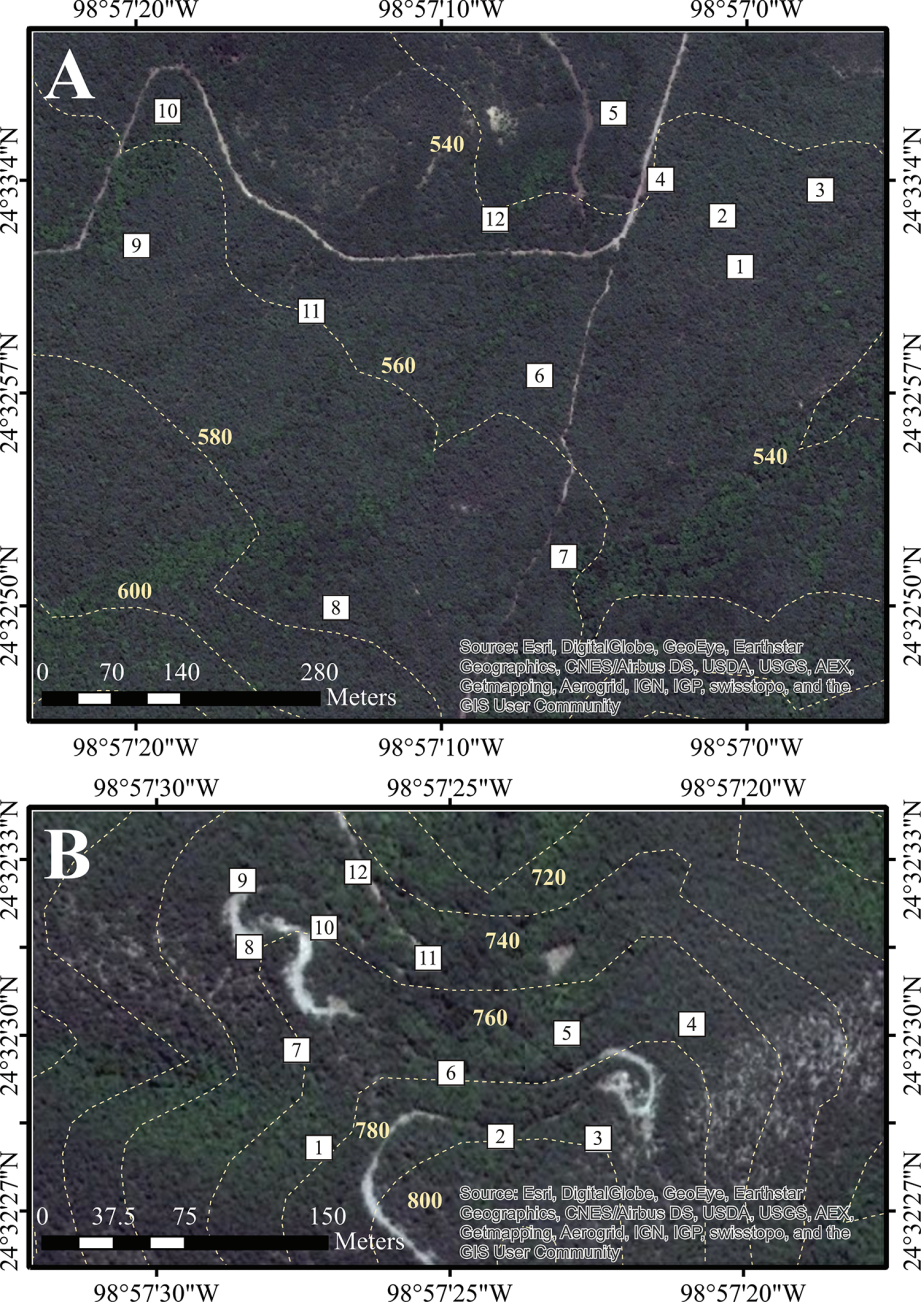
Detailed position of sampling plots in Sierra de San Carlos. Cerro El Diente locality: **A** Site 1 **B** Site 2. Dotted lines shows elevation curves.

**Figure 3.**
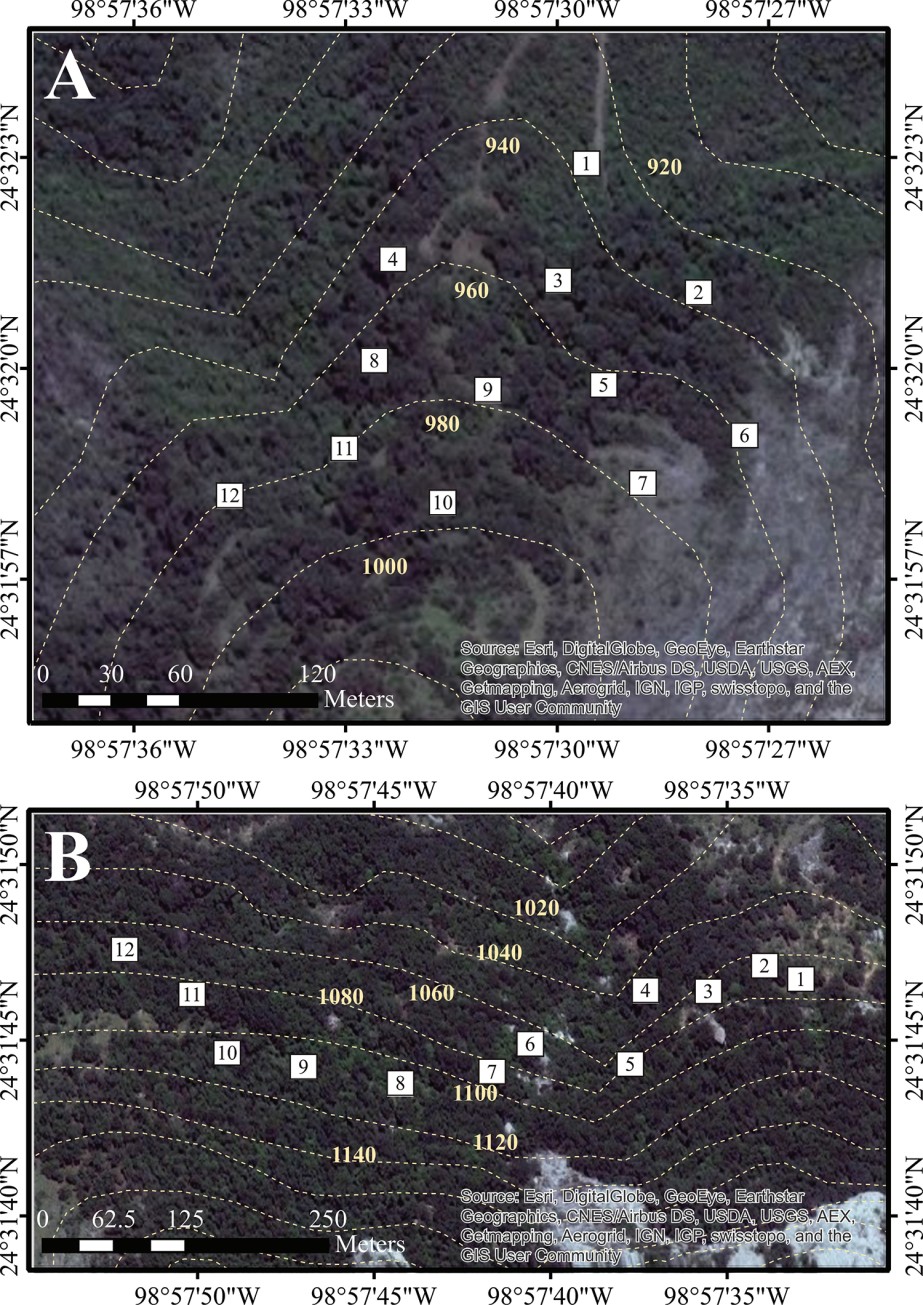
Detailed position of sampling plots in Sierra de San Carlos. Cerro El Diente locality: **A** Site 3 **B** Site 4. Dotted lines shows elevation curves.

**Figure 4.**
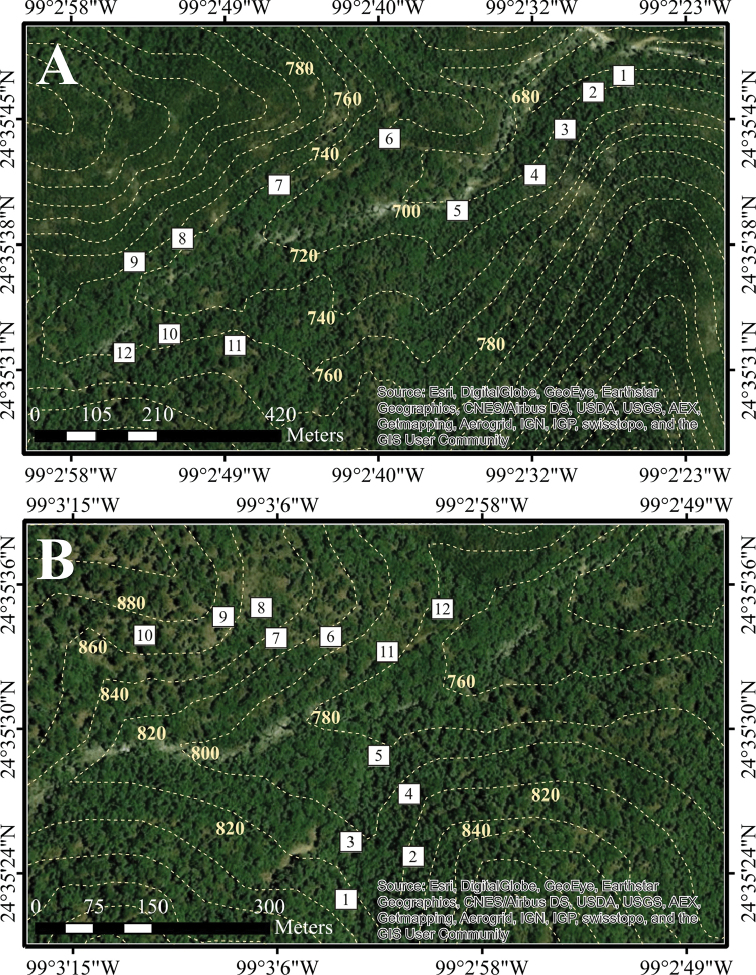
Detailed position of sampling plots in Sierra de San Carlos. Ejido Carricitos y Tinajas locality: **A** Site 5 **B** Site 6. Dotted lines shows elevation curves.

**Figure 5.**
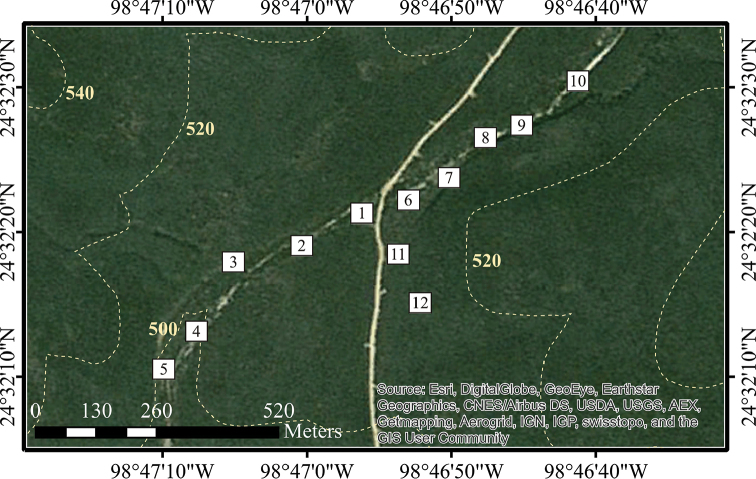
Detailed position of sampling plots in Sierra de San Carlos. San Nicolás locality, Site 7. Dotted lines shows elevation curves.

Twelve sampling plots of 400 m^2^ each (20 × 20 m) were established within each of the seven sampling sites. Plot dimensions were determined with the species-area curve method, using the nested quadrat type (Scheiner 2003). The number of plots sampled was established by Clench analysis, with 70% as a minimum limit of completeness (for a detailed analysis, see Jiménez-Valverde and Hortal 2003). The plots were previously and randomly located within each of these sites, using GIS software. This was accomplished by: 1) creating a polygon shape for the selected area of the sampling site, and 2) delimiting a square graticule inside this polygon using the Repeating Shapes tool, with the sides of the squares 20 meters in length; both procedures were made using ArcView GIS (1992–1999). 3) The graticule was exported to IDRISI Selva (1987-2012) and converted to a .kml format archive; 4) the new archive was displayed in Google Earth Pro software, permitting the visualization of the graticule of real dimension plots displayed over actual satellite imagery; and finally, 5) this graticule was treated as a coordinate system (rows and columns), and the location of each one of the 12 plots was selected by the random numbers tool in Microsoft Office Excel. These methods were employed for the seven sites, and the only difference was the dimensions of the polygon shape of the sample area which were adjusted to the vegetational communities. Plots were georeferenced and later located in the field. When a plot was located in areas not accessible or impossible to sample (i.e. steep hills, areas of bare soil), it was moved to the closest location where vegetational cover was available. Detailed geographical data (latitude and longitude coordinates, elevation) for each plot within each site are presented in Tables 1–3; detailed spatial arrangement of sampling plots within each site is presented in Figures 2–5.

**Table 1.** Sampling data in the Cerro El Diente locality, Sierra de San Carlos, Mexico (coordinates at center of plot; elevation in meters).

Cerro El Diente							
Site 1 – Submountain scrub				Site 2 – Tamaulipan thorny scrub			
Sampling plot	Latitude	Longitude	Elevation	Sampling plot	Latitude	Longitude	Elevation
P1	24°33.020'N	98°57.004'W	550	P1	24°32.468'N	98°57.454'W	772
P2	24°33.048'N	98°57.013'W	548	P2	24°32.471'N	98°57.402'W	790
P3	24°33.062'N	98°56.960'W	544	P3	24°32.471'N	98°57.374'W	784
P4	24°33.068'N	98°57.047'W	547	P4	24°32.492'N	98°57.353'W	766
P5	24°33.104'N	98°57.073'W	535	P5	24°32.501'N	98°57.383'W	773
P6	24°32.996'N	98°57.096'W	555	P6	24°32.490'N	98°57.416'W	778
P7	24°32.936'N	98°57.082'W	561	P7	24°32.496'N	98°57.460'W	760
P8	24°32.920'N	98°57.173'W	570	P8	24°32.522'N	98°57.469'W	750
P9	24°33.031'N	98°57.333'W	571	P9	24°32.537'N	98°57.473'W	743
P10	24°33.105'N	98°57.316'W	557	P10	24°32.531'N	98°57.452'W	750
P11	24°32.995'N	98°57.237'W	560	P11	24°32.522'N	98°57.423'W	755
P12	24°33.046'N	98°57.137'W	540	P12	24°32.543'N	98°57.441'W	745
Site 3 – Oak forest				Site 4 – Cloud forest			
Sampling plot	Latitude	Longitude	Elevation	Sampling plot	Latitude	Longitude	Elevation
P1	24°32.038'N	98°57.496'W	938	P1	24°31.780'N	98°57.557'W	1077
P2	24°32.018'N	98°57.466'W	935	P2	24°31.795'N	98°57.565'W	1065
P3	24°32.021'N	98°57.500'W	950	P3	24°31.780'N	98°57.593'W	1070
P4	24°32.026'N	98°57.539'W	948	P4	24°31.774'N	98°57.622'W	1055
P5	24°31.996'N	98°57.489'W	964	P5	24°31.753'N	98°57.631'W	1085
P6	24°31.984'N	98°57.456'W	948	P6	24°31.760'N	98°57.672'W	1077
P7	24°31.973'N	98°57.480'W	971	P7	24°31.744'N	98°57.695'W	1093
P8	24°32.002'N	98°57.543'W	967	P8	24°31.730'N	98°57.738'W	1112
P9	24°31.995'N	98°57.516'W	974	P9	24°31.738'N	98°57.783'W	1109
P10	24°31.968'N	98°57.527'W	993	P10	24°31.751'N	98°57.816'W	1102
P11	24°31.981'N	98°57.550'W	982	P11	24°31.776'N	98°57.831'W	1086
P12	24°31.970'N	98°57.577'W	979	P12	24°31.793'N	98°57.868'W	1076

**Table 2.** Sampling data in the Ejido Carricitos y Tinajas locality, Sierra de San Carlos, Mexico (coordinates at center of plot; elevation in meters).

Ejido Carricitos y Tinajas							
Site 5 – Riparian and secondary vegetation				Site 6 – Oak and pine forests			
Sampling plot	Latitude	Longitude	Elevation	Sampling plot	Latitude	Longitude	Elevation
P1	24°35.807'N	99°2.450'W	700	P1	24°35.397'N	99°3.037'W	839
P2	24°35.789'N	99°2.484'W	701	P2	24°35.420'N	99°3.023'W	830
P3	24°35.764'N	99°2.508'W	704	P3	24°35.440'N	99°3.041'W	816
P4	24°35.727'N	99°2.534'W	712	P4	24°35.463'N	99°3.017'W	814
P5	24°35.684'N	99°2.600'W	716	P5	24°35.491'N	99°3.028'W	795
P6	24°35.719'N	99°2.654'W	720	P6	24°35.567'N	99°3.067'W	813
P7	24°35.673'N	99°2.766'W	740	P7	24°35.563'N	99°3.101'W	827
P8	24°35.632'N	99°2.851'W	757	P8	24°35.575'N	99°3.124'W	846
P9	24°35.605'N	99°2.894'W	764	P9	24°35.579'N	99°3.146'W	860
P10	24°35.571'N	99°2.870'W	763	P10	24°35.577'N	99°3.175'W	866
P11	24°35.545'N	99°2.806'W	773	P11	24°35.548'N	99°3.013'W	788
P12	24°35.533'N	99°2.909'W	776	P12	24°35.584'N	99°2.970'W	780

**Table 3.** Sampling data in the San Nicolás locality, Sierra de San Carlos, Mexico (coordinates at center of plot; elevation in meters).

Site 7 – Tamaulipan thorny scrub and submountain scrub vegetation			
Sampling plot	Latitude	Longitude	Elevation
P1	24°32.356'N	98°46.936'W	502
P2	24°32.319'N	98°47.006'W	501
P3	24°32.290'N	98°47.073'W	500
P4	24°32.224'N	98°47.117'W	499
P5	24°32.180'N	98°47.153'W	499
P6	24°32.371'N	98°46.883'W	503
P7	24°32.391'N	98°46.840'W	508
P8	24°32.427'N	98°46.797'W	510
P9	24°32.450'N	98°46.759'W	508
P10	24°32.485'N	98°46.702'W	508
P11	24°32.334'N	98°46.922'W	502
P12	24°32.295'N	98°46.906'W	503

### Collection and processing of specimens

Systematic sampling was conducted between 10:00 and 17:00 h, using a standard entomological sweep net of 40 cm diameter. Individual samples consisted of 120-200 sweeps of the shrub and herbaceous vegetation in each plot. Contents of the net were emptied into a plastic bag, adding 60% ethanol and an indelible label with corresponding data. Each plot (12) within the seven sites was sampled monthly, from February 2013 to January 2014, comprising 1,008 total samples at the end of the study; sweeping was conducted by the same person during the whole study to reduce sampling error. Each sample and the specimens obtained were processed according to the method described by Sánchez-Reyes et al. (2014). Also, leaf beetles encountered independent of the standardized sweeps were added to the species checklist. Specimens are deposited in the collection of the Facultad de Ingeniería y Ciencias at the Universidad Autónoma de Tamaulipas, Ciudad Victoria, Tamaulipas, Mexico.

### Taxonomic determination

Identification of specimens was made using available literature on Chrysomelidae (Wilcox 1965, White 1968, Wilcox 1972, Scherer 1983, White 1993, Flowers 1996, Riley et al. 2002, Staines 2002). Additionally, material was compared with identified specimens deposited in the collection of Chrysomelidae of the Facultad de Ingeniería y Ciencias, Universidad Autónoma de Tamaulipas. However, those specimens that could not be identified to the species level were compared with other unidentified specimens and carefully grouped into morphospecies, and so the designation of “species” in this study includes both morphospecies and determined species. Taxonomical arrangement follows the categories employed by Riley et al. (2003), except that the subfamily Bruchinae is not included in this study.

### Organization of seasonal data

We obtained environmental data from two meteorological stations located in the municipalities of San Carlos and San Nicolás in the study area. Historical data of total monthly rainfall and monthly average temperature (only the average from 1951-2010 was available) were plotted to visually analyze the fluctuation of these parameters. On this basis, four seasons were defined: Early dry season (EDS: November, December, January), Late dry season (LDS: February, March, April), Early rainy season (ERS: May, June, July), and Late rainy season (LRS: August, September, October). Data of precipitation and temperature were correlated with species richness and abundance, using a Spearman correlation analysis in STATISTICA 8.0 (StatSoft Inc. 2007).

### Data analysis

All the following analyses were made only with the data obtained through systematic sampling (i.e., by sweeping in 12 plots at each site). Species collected otherwise were excluded from the analysis, but are included in the checklist of species.

As a measure of species richness, we used the total number of species present throughout the Sierra de San Carlos, and at each site and season. Significant differences in the number of species were assessed through permutation tests in PAST 3.07 (Hammer et al. 2001). Estimated species richness at each level was measured by means of the nonparametric estimators ACE, Chao 1, and Jackknife 1 (Magurran 2004, Hortal et al. 2006, Gotelli and Colwell 2011), and was calculated with the software EstimateS 8.2 (Colwell 2013), using 100 randomizations without replacement and the abundance data of each species found per sampling unit (plot). Also, we used the Clench model to determine the sampling efficiency through the estimated species richness and the slope of the species accumulation curve, which measures the inventory quality; calculations were made according to the method described by Jiménez-Valverde and Hortal (2003), and were performed in STATISTICA 8.0 (StatSoft Inc. 2007).

Species from Sierra de San Carlos were divided into five categories, according to their total abundance: 1) very common (more than 70 individuals); 2) common (11 to 70 specimens); 3) rare (3 to 10 specimens); 4) doubletons (two specimens); and 5) singletons (one specimen only). These categories were used because they have been implemented in similar studies with Chrysomelidae, also in the state of Tamaulipas (Sánchez-Reyes et al. 2014). Also, abundance was measured at each site and season, and differences between these values were analyzed with Kruskal-Wallis and Mann-Whitney nonparametric tests, as data did not meet the assumptions of normality. We used the Simpson dominance index (D) and the Shannon entropy index (H’) to measure both principal components of alpha diversity (Magurran 2004). Values obtained were transformed to a true diversity value according to Jost (2006, 2007), and were calculated for the whole study area and for each site and season. Differences in species composition between each pairwise comparison of sites and months were assessed through PERMANOVA analysis, using the Bray-Curtis index as distance measure, with 9999 random permutations. Beta diversity was measured as the faunistic similarity between sites and months, using the Bray-Curtis index of similarity; also, a Cluster analysis was performed to define groups of sites and months according to species composition, using the Bray-Curtis index as a distance measure and the Ward method as an amalgamation algorithm. All calculations were made in PAST 3.07 (Hammer et al. 2001) and STATISTICA 8.0 (StatSoft Inc. 2007).

Finally, the association of abundance and species richness obtained at each elevational site during each month was measured with a Correspondence analysis. This is a multivariate technique based on contingency tables and count data, where the significant statistical dependence between rows (sites) and columns (months) is tested by a chi-square test (Beh 2004). The analysis was conducted in STATISTICA 8.0.

## Results

### Leaf beetles in Sierra de San Carlos

**Figure 6.**
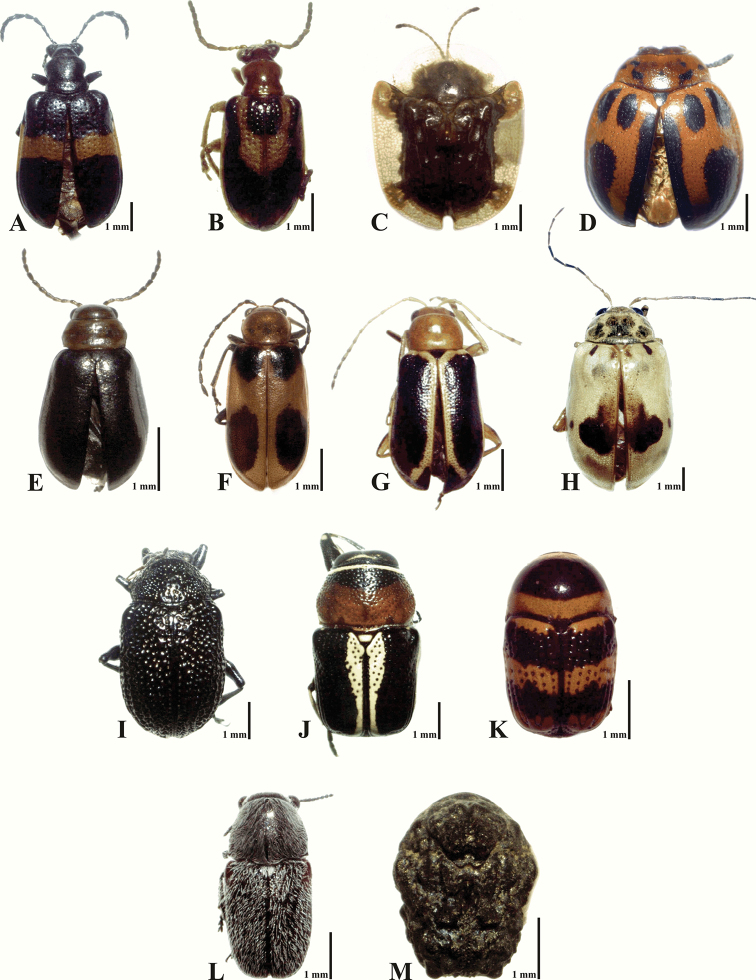
Examples of leaf beetle biodiversity from Sierra de San Carlos, Mexico. **A** *Lema balteata* LeConte, 1884 **B** *Lema opulenta* Harold, 1874 **C** *Helocassis clavata* (Fabricius, 1798) **D** *Plagiodera semivittata* Stål, 1860 **E** *Miraces aeneipennis* Jacoby, 1888 **F** *Malacorhinus acaciae* (Schaeffer, 1906)**G** *Cyclotrypema furcata* (Olivier, 1808) **H** *Acrocyum dorsalis* Jacoby, 1885 **I** *Colaspis melancholica* Jacoby, 1881 **J** *Griburius montezuma* (Suffrian, 1852) **K** *Cryptocephalus trizonatus* Suffrian, 1858 **L** *Coscinoptera tamaulipasi* Medvedev, 2012 **M** *Diplacaspis prosternalis* (Schaeffer, 1906).

**Figure 7.**
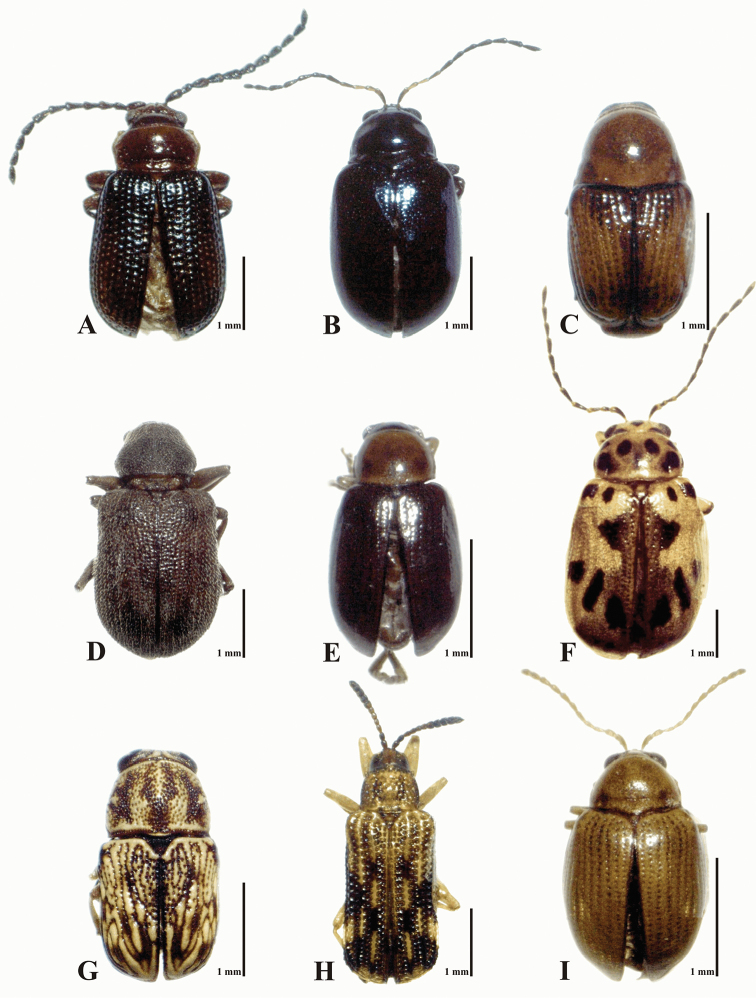
Examples of leaf beetle biodiversity from Sierra de San Carlos, Mexico. **A** *Trichaltica scabricula* (Crotch, 1873), new country record. In decreasing order from **B** to **I**, the most abundant species in the current study. **B** *Syphrea* sp. 2 **C** *Diachus* sp. 1 **D** *Xanthonia* sp. 1 **E** *Centralaphthona diversa* (Baly, 1877) **F** *Chrysogramma* sp. 1 **G** *Pachybrachis* sp. 1 **H** *Sumitrosis inaequalis* (Weber, 1801) **I** *Margaridisa atriventris* (Melsheimer, 1847).

In total, 3,081 specimens, belonging to 109 species, 63 genera and six subfamilies, were obtained. An additional four species were obtained by independent collecting in various sites from study area, resulting in a total species richness of 113 species and 65 genera within the study period (2013-2014) in the Sierra de San Carlos (Appendix 1, Figures 6–7). According to ACE, Chao 1, and Jackknife indexes, the estimated species richness was between 115.88 and 128.98 species; also, the estimation using the Clench Model was 120 species. These values indicate that the proportion of observed species richness in relation to the richness estimates was 84.5 to 94% (Table 4). Slope value of the Clench Model suggests that faunistic inventory in Sierra de San Carlos was complete and reliable (slope = 0.012). The Dominance value for the Simpson index (D) was 0.066, with a diversity value (1/D) of 15.064. For the Shannon index (H´), the value was 3.41, with a diversity value (e*^H^*^´^) of 30.29.

**Table 4.** Observed and estimated species richness of Chrysomelidae by site and season at Sierra de San Carlos, Mexico. Elevation in meters. EDS = Early dry season, LDS = Late dry season, ERS = Early rainy season, LRS = Late rainy season.

	S*_obs_*	Nonparametric indexes			Clench model		Completeness (%)
		Chao 1	Jack 1	ACE	S*_est_*	Slope	
Elevation*							
500 (S7)	33 d	36.5±3.5	42.93±3.35	38.7±0.65	42.07	0.05	76.8–90.4
550 (S1)	38 abcd	41.57±3.1	48.92±3.75	47.58±0	53.82	0.07	77.6–91.4
730 (S5)	36 defg	52.9±12.72	49.9±4.06	50.57±0.71	48.31	0.066	68.05–72.1
760 (S2)	40 ae	40.89±1.26	46.95±2.57	43.43±0.46	50.51	0.05	85.19–97.82
820 (S6)	27 cg	39.25±13.15	33.95±2.93	33.62±0.71	31.47	0.03	68.78–79.52
960 (S3)	47 bf	50.38±3.06	56.93±3.35	52.83±0.53	60.83	0.074	82.55–93.29
1080 (S4)	50 b	58.64±6.82	62.91±5.44	58.76±0.49	60.89	0.065	79.47–85.26
Season*							
EDS	40 a	44±3.74	48.96±2.94	45.88±0.5	47.5	0.026	81.69–90.9
LDS	49 b	55.4±5.92	57.96±3.26	53.55±0.27	57.49	0.029	84.54–88.44
ERS	78 c	91.88±8.32	102.9±5.69	94.66±1.17	102.17	0.075	75.8–84.89
LRS	76 c	82.62±4.57	93.93±4.54	87.65±0.77	92.97	0.058	80.91–91.98
**Total**	109	115.88±4.5	128.98±5.06	122.47±0	120.46	0.012	84.5–94

S*_obs_* = observed species richness; S*_est_* = estimated species richness. *Species richness values of sites and seasons with different letters between rows are significantly different from each other (*p*<0.05), according to permutation tests.

The most abundant subfamily in the study area was Galerucinae (including Alticini), with 53.6% (1,652 specimens) of the total abundance of Chrysomelidae in the Sierra de San Carlos, followed by Cryptocephalinae with 26.9% (828 specimens). Less abundance was found in Eumolpinae (12.6%, 388 specimens), Cassidinae (4.9%, 152 specimens), Chrysomelinae (1.1%, 33 specimens) and Criocerinae (0.9%, 28 specimens). Species counts per subfamily were greatest for Galerucinae, with 54 species (49.5%) representing half of the total species richness recorded in Sierra de San Carlos. Lower values were recorded for Cryptocephalinae with 27 species (24.8%), Eumolpinae with nine species (8.3%), Cassidinae and Criocerinae both with eight species (7.3%), and Chrysomelinae with only three species (2.8%).

Eight species were categorized as “very abundant species,” each with over 70 specimens that accounted for 60.3% (1,859 total specimens) of the total abundance obtained from Sierra de San Carlos. Of these eight species, *Syphrea* sp. 2 (475 specimens) and *Diachus* sp. 1 (418) were the most abundant, followed by *Xanthonia* sp. 1 (276), *Centralaphthona diversa* (Baly, 1877) (244), *Chrysogramma* sp. 1 (193), *Pachybrachis* sp. 1 (103), *Sumitrosis inaequalis* (Weber, 1801) (78), and *Margaridisa atriventris* (Melsheimer, 1847) (72) (Figure 7B–7I). The proportion of 31.6% (974 specimens) of the total abundance was accounted for by 34 species categorized as “common,” while 29 species were considered as “rare” (6.13% of the total abundance). Only 21 species were doubletons, and 17 were singletons. *Trichaltica scabricula* (Crotch, 1873) is recorded for the first time in Mexico (Figure 7A).

### Species richness and inventory completeness of Chrysomelidae by elevation site and season in Sierra de San Carlos

No clear patterns of species richness were found with elevation. The greatest number of species (50) was recorded at the site of highest elevation (1080 masl), but this value was not significantly different from values observed at 960 (47 species) and 550 masl (38 species). The smallest number of species (27) was registered at a high elevation site (820 masl); however, it was not significantly different from sites at 730 (36 species) and 550 masl (Table 2). Completeness exceeded 70% in all sites, with a maximum value of 97% at Site 2 (760 masl), although lower values were obtained at both sites from the Carricitos y Tinajas locality (730 and 820 masl). The slope of the Clench model was less than 0.1 for each of the sites (Table 4).

Regarding seasonal analysis, the species richness increased progressively and significantly from early dry season (40 species) to early rainy season (78 species). The value decreased to 76 species during late rainy season, although this change was not significant. Seasonal values of estimated species richness through nonparametric indexes and the Clench model followed the same pattern as that of sites, because all values were above 70% of completeness, with slopes under 0.1 for all the seasons (Table 4).

### Elevational patterns of leaf beetle abundance and diversity in Sierra de San Carlos

We found significant variations in abundance and diversity of Chrysomelidae between sites of differing elevation (Kruskal Wallis Test, H=100.7, *p*<0.0001). However, these parameters did not show a specific trend with the increase or decrease in elevation. For example, the highest abundance (665 individuals) was present at the lowest site (500 masl), whereas the lowest value (173 individuals) was obtained at the second lowest elevation (550 masl). Also, differences in abundance obtained between the site of lowest elevation (665 individuals) and site at 960 masl (561 individuals) were not significant, while the number of specimens (440) at the highest site was not statistically different from values observed at lower sites (960, 820 and 760 masl). The lowest abundances were present at 550 (173 specimens) and 730 masl (231 specimens), and these values were significantly different from other elevational sites (Tables 5, 6).

**Table 5.** Mann-Whitney pairwise comparisons of chrysomelid abundance between elevational sites in Sierra de San Carlos, Mexico. Upper diagonal = Mann-Whitney U values. Lower diagonal = *p* values; marked values (*) are significant.

	S1–550 m	S2–760 m	S3–960 m	S4–1080 m	S5–730 m	S6–820 m	S7–500 m
**Site 1**	–	6723	5344	6289	8234	6197	4675
**Site 2**	<0.0001*	–	8853	9920	8336	9735	8126
**Site 3**	<0.0001*	0.03007*	–	9232	6782	9509	9634
**Site 4**	<0.0001*	0.52	0.1042	–	7778	10100	8530
**Site 5**	0.001504*	0.003257*	<0.0001*	0.000181*	–	7707	6002
**Site 6**	<0.0001*	0.3634	0.2197	0.748	0.000121*	–	8758
**Site 7**	<0.0001*	0.001351*	0.2958	0.008679*	<0.0001*	0.02159*	–

As observed with abundance, no clear patterns of diversity were found with elevation, although all sites were significantly different from each other. The highest dominance (D=0.406) and lowest entropy (H’=1.716) were obtained in the site of lowest elevation (500 m), indicating that the lowest diversity was found at this site (1/D=2.46; e*^H’^*=5.56). However, the second highest value of diversity in the study area (1/D=12.66; and e*^H’^*=21.47) was obtained at the second lowest elevation site (550 meters). Diversity decreased progressively from the third (730 meters) to the fifth elevational site (820 m), and then increased from 960 masl to the highest elevation site (1080 masl), where the lowest dominance (D=0.0508) and highest values of entropy (H’=3.328) and diversity (1/D=19.66; e*^H’^*=27.88) were obtained (Table 6).

**Table 6.** Elevational variation of abundance and diversity of Chrysomelidae in Sierra de San Carlos, Mexico.

Parameter	Study site ^‡^						
	S7	S1	S5	S2	S6	S3	S4
Elevation (masl)	500	550	730	760	820	960	1080
Abundance ^†^	665 a	173 b	231 c	432 d	579 df	561 aef	440 de
**Diversity** *							
D = Simpson index (dominance)	0.406 a	0.078 b	0.101 c	0.137 d	0.183 e	0.156 f	0.0508 g
1/D = Simpson Diversity index	2.46 a	12.66 b	9.89 c	7.29 d	5.45 e	6.38 f	19.66 g
H´ = Shannon index	1.716 a	3.067 b	2.819 c	2.638 d	2.185 e	2.576 d	3.328 g
*e* ^H´^ = Shannon Diversity	5.56 a	21.47 b	16.76 c	13.98 d	8.89 e	13.14 d	27.88 g

^‡^S1 to S7 = Sites 1 to 7, arranged from low to high elevation. Details about the numbering of the sites are in the Materials and Methods section. ^†^ Abundance values with different letters between columns are significantly different from each other (Kruskal-Wallis, H=100.7, *p*=0.000), according to *p* values in Table 5. *Diversity values with different letters between columns are significantly different from each other (*p*<0.05), according to permutation tests.

**Figure 8.**
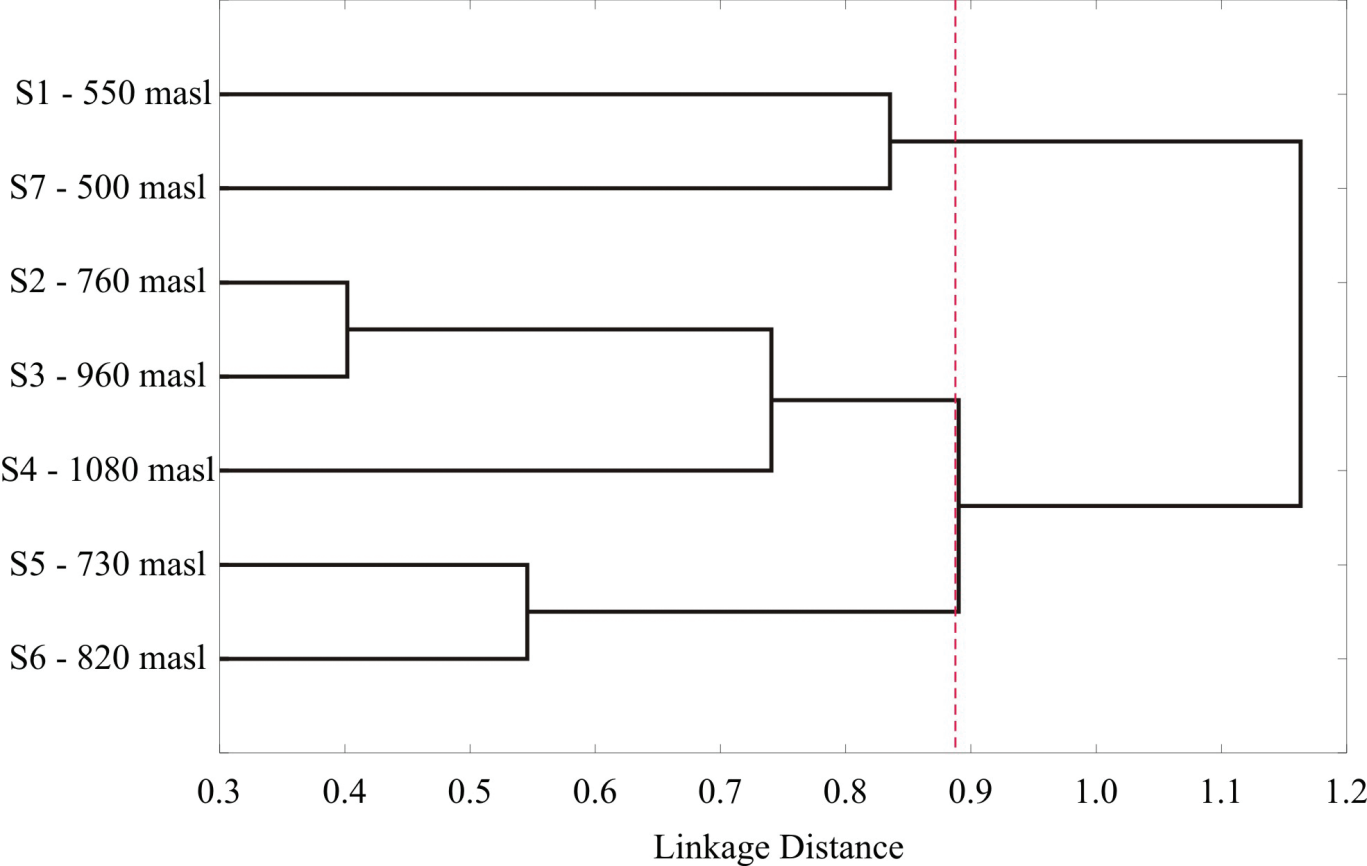
Cluster analysis of chrysomelid composition by elevational site in Sierra de San Carlos, Mexico. Delimitation of groups is indicated by red dotted line.

According to the PERMANOVA analysis, the leaf beetle composition between sites was statistically different (SS_total_=33.16; SS_within-group_=23.22; F=5.492, *p*=0.0001), and almost all pairwise comparisons were significantly different, except for Site 2 (760 masl) and Site 3 (960 masl) (F=1.595, *p*=0.1151) (Table 7). The Bray-Curtis index also showed that Site 2 and Site 3 were the most similar in the study area, with 59% faunistic similarity. Values below 50% were obtained for all other comparisons, with the lowest percentages associated with Site 7 when compared to all others (Table 8). Cluster analysis suggested that three groups were generated on the basis of the difference in leaf beetle composition between elevation sites: Site 5 and Site 6 (Group 1); Site 2, Site 3 and Site 4 (Group 2); Site 1 and Site 7 (Group 3) (Figure 8).

**Table 7.** PERMANOVA pairwise comparisons of chrysomelid composition between elevational sites in Sierra de San Carlos, Mexico. Upper diagonal = F values. Lower diagonal = *p* values.

	S7–500 m	S1–550 m	S5–730 m	S2–760 m	S6–820 m	S3–960 m	S4–1080 m
**Site 7**	–	7.446	11.65	10.89	12.17	9.959	9.959
**Site 1**	0.0001	–	4.59	3.452	5.391	4.057	4.692
**Site 5**	0.0001	0.0001	–	5.13	2.497	4.229	4.658
**Site 2**	0.0001	0.0001	0.0001	–	4.171	1.595	3.449
**Site 6**	0.0001	0.0001	0.0182	0.0001	–	3.231	3.636
**Site 3**	0.0001	0.0001	0.0001	0.1151	0.0002	–	2.644
**Site 4**	0.0001	0.0001	0.0001	0.0001	0.0001	0.0007	–

**Table 8.** Bray–Curtis similarity between elevational sites in Sierra de San Carlos, Mexico. Upper diagonal = Index values. Lower diagonal = values expressed as percentage of similarity.

	S1–550 m	S2–760 m	S3–960 m	S4–1080 m	S5–730 m	S6–820 m	S7–500 m
**Site 1**	–	0.25455	0.14441	0.16639	0.24752	0.16755	0.16468
**Site 2**	25.455	–	0.59819	0.31651	0.19005	0.36993	0.074749
**Site 3**	14.441	59.819	–	0.37163	0.18434	0.45965	0.03752
**Site 4**	16.639	31.651	37.163	–	0.27422	0.33562	0.050679
**Site 5**	24.752	19.005	18.434	27.422	–	0.45432	0.051339
**Site 6**	16.755	36.993	45.965	33.562	45.432	–	0.067524
**Site 7**	16.468	7.4749	3.752	5.0679	5.1339	6.7524	–

### Seasonal patterns of leaf beetle abundance and diversity in Sierra de San Carlos

**Figure 9.**
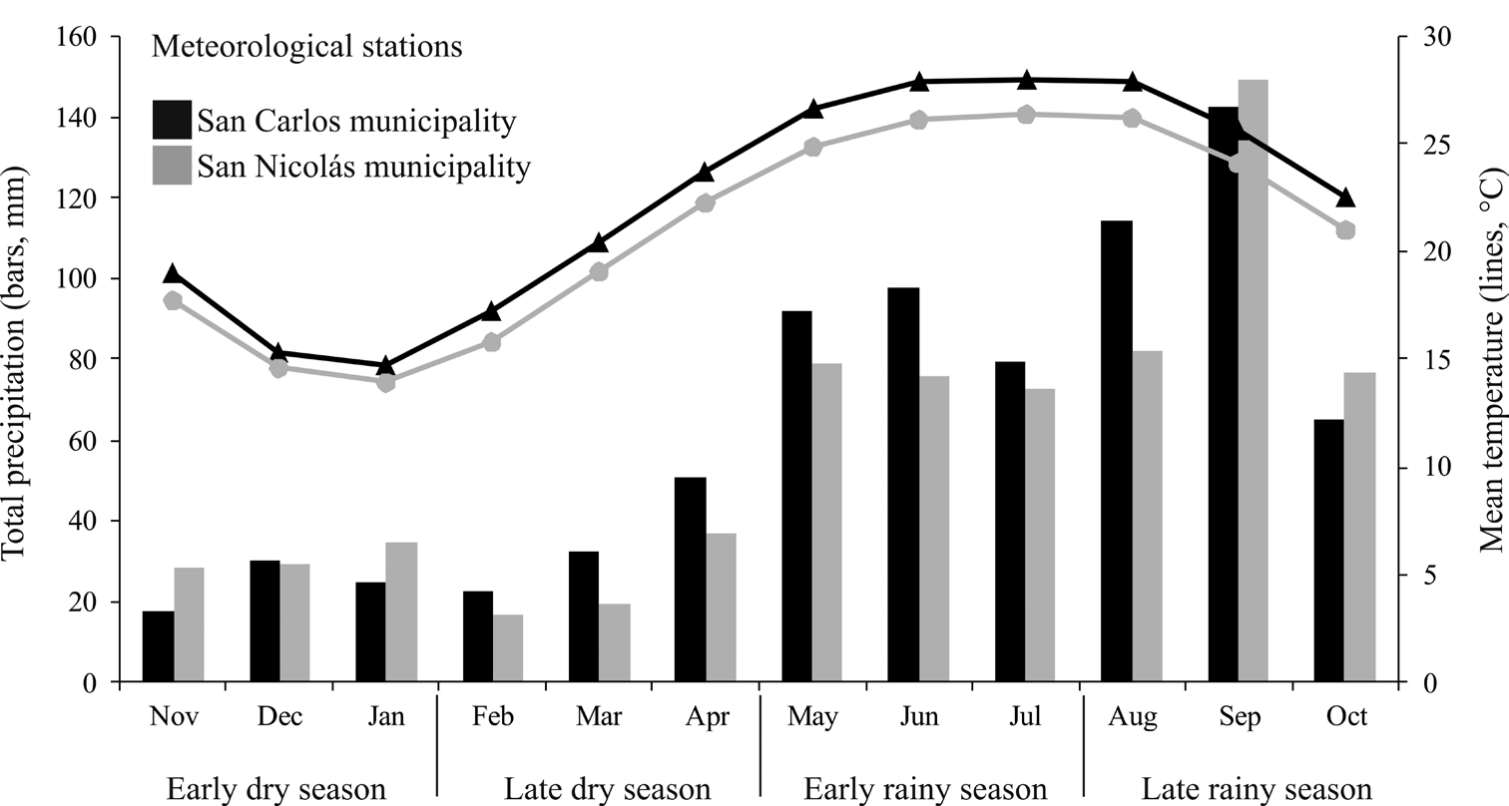
Historical monthly data of precipitation and temperature within Sierra de San Carlos, Mexico.

Abundance and diversity values showed significant variation between seasons (H=92.29, *p*<0.0001). The lowest number of specimens (433) was recorded at early dry season, and abundance increased significantly at the end of the season (888 specimens). Abundance decreased at early rainy season (690 specimens), although this reduction was not significant; then, the value increased significantly at the late rainy season, which recorded the highest abundance (1,070 specimens) in this study (Tables 9, 10). Similarly, the highest dominance (D=0.146) and the lowest values of entropy (H’=2.736) and diversity (1/D=6.84; H’=15.42) were obtained in the early dry season. These values changed significantly at late dry season (1/D=9.90; H’=17.01) and also during the early rainy season, when the highest diversity was obtained (1/D =18.68; H’=33.38) (Table 10). Monthly temperature and precipitation data (Figure 9) showed a significant positive correlation (*p*<0.05) with the number of species recorded by month in Sierra de San Carlos, whereas abundance was not correlated with environmental parameters (Table 11).

**Table 9.** Mann-Whitney pairwise comparisons for chrysomelid abundance between seasons in Sierra de San Carlos, Mexico. Upper diagonal = Mann-Whitney U values. Lower diagonal = *p* values; marked values (*) are significant.

	Early dry season	Late dry season	Early rainy season	Late rainy season
Early dry season	–	22600	23100	16400
Late dry season	<0.0001*	–	30900	25400
Early rainy season	<0.0001*	0.597	–	24300
Late rainy season	<0.0001*	0.000083*	0.0000038*	–

**Table 10.** Seasonal variation of abundance and diversity of Chrysomelidae in Sierra de San Carlos, Mexico.

Parameter	Dry Season		Rainy Season	
	Early (EDS)	Late (LDS)	Early (ERS)	Late (LRS)
**Abundance** ^‡^	433 a	888 b	690 b	1070 c
Diversity*				
D = Simpson index (dominance)	0.146 a	0.101 b	0.053 c	0.104 b
1/D = Simpson Diversity index	6.84 a	9.90 b	18.68 c	9.59 b
H´ = Shannon index	2.736 a	2.834 b	3.508 c	3.091 d
*e* ^H´^ = Shannon Diversity	15.42 a	17.01 b	33.38 c	21.99 d

‡ Abundance values with different letters between columns are significantly different from each other (Kruskal-Wallis, H=92.29, *p*=0.000), according to *p* values in Table 9. *Diversity values with different letters between columns are significantly different from each other (p<0.05), according to permutation tests.

**Table 11.** Spearman correlation analysis for abundance and species richness of Chrysomelidae with temperature and precipitation in Sierra de San Carlos, Mexico. Marked values (*) are significant.

	Temperature [°C]		Precipitation [mm]	
	**San Carlos**	**San Nicolás**	**San Carlos**	**San Nicolás**
Abundance	0.532	0.559	0.531	0.349
Species Richness	0.809*	0.822*	0.826*	0.710*

**Figure 10.**
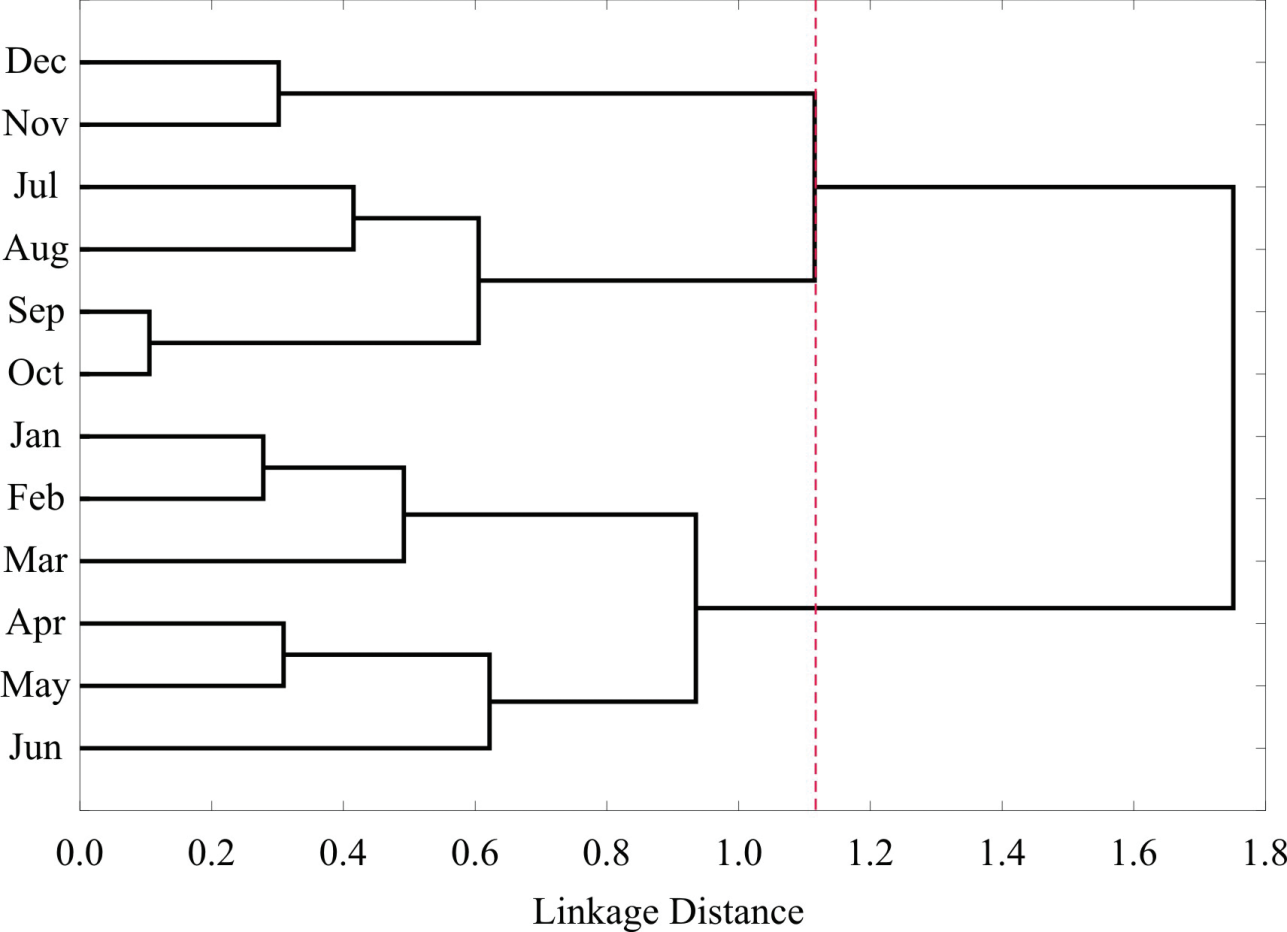
Cluster analysis of chrysomelid composition by month in Sierra de San Carlos, Mexico. Delimitation of groups is indicated by red dotted line.

Seasonal species composition and faunistic similarity were analyzed using monthly values. Significant differences in species composition between months were found (SS_total_=33.16; SS_within-group_=27.33; F=1.395, p=0.0009). Pairwise comparisons showed that differences were obtained principally between early (January, February, March, April and May) and later months (July, September, October, November and December) (Table 12). The highest similarity occurred between September-October (89.4%), January-February (72.1%), November-December (69.8%), April-May (69%), and February-March (63.6%), whereas other comparisons were less than or close to 50% (Table 13). Three clusters were formed according to monthly leaf beetle composition: January to June (Group 1), July to October (Group 2), and November-December (Group 3) (Figure 10).

**Table 12.** PERMANOVA pairwise comparisons of chrysomelid composition between months in Sierra de San Carlos, Mexico. Upper diagonal = F values. Lower diagonal = *p* values; values in bold are significant.

	**Jan**	**Feb**	**Mar**	**Apr**	**May**	**Jun**	**Jul**	**Aug**	**Sep**	**Oct**	**Nov**	**Dec**
**Jan**	–	0.964	0.344	0.063	0.105	0.312	**0.029**	0.076	**0.017**	**0.022**	**0.010**	**0.031**
**Feb**	0.191	–	0.799	0.055	0.093	0.299	**0.040**	0.162	**0.036**	**0.029**	**0.014**	**0.039**
**Mar**	1.076	0.629	–	0.666	0.350	0.115	**0.031**	0.179	**0.023**	**0.014**	**0.010**	**0.033**
**Apr**	1.668	1.583	0.812	–	0.939	0.206	**0.038**	0.075	**0.009**	**0.004**	**0.012**	**0.047**
**May**	1.442	1.407	1.076	0.493	–	0.587	0.287	0.163	**0.008**	**0.006**	**0.014**	**0.035**
**Jun**	1.147	1.168	1.416	1.23	0.902	–	0.467	0.685	0.083	0.082	**0.005**	**0.013**
**Jul**	1.749	1.607	1.639	1.563	1.12	0.995	–	0.879	0.546	0.479	0.071	0.078
**Aug**	1.566	1.33	1.34	1.495	1.264	0.844	0.638	–	0.739	0.627	**0.012**	**0.032**
**Sep**	1.89	1.713	1.819	1.973	1.786	1.459	0.933	0.709	–	0.962	0.107	0.088
**Oct**	1.89	1.8	1.988	2.08	1.831	1.486	0.986	0.821	0.041	–	0.078	0.101
**Nov**	1.916	1.809	1.722	1.718	1.778	1.929	1.491	1.864	1.544	1.624	–	0.972
**Dec**	1.678	1.607	1.562	1.448	1.552	1.725	1.461	1.672	1.502	1.528	0.106	–

**Table 13.** Bray-Curtis similarity between months in Sierra de San Carlos, Mexico. Upper diagonal = Index values. Lower diagonal = values expressed as percentage of similarity.

	**Jan**	**Feb**	**Mar**	**Apr**	**May**	**Jun**	**Jul**	**Aug**	**Sep**	**Oct**	**Nov**	**Dec**
**Jan**	-	0.721	0.487	0.36	0.398	0.433	0.298	0.356	0.295	0.309	0.230	0.273
**Feb**	72.1	-	0.636	0.347	0.35	0.367	0.313	0.390	0.309	0.318	0.203	0.215
**Mar**	48.7	63.6	-	0.563	0.482	0.340	0.319	0.401	0.353	0.365	0.161	0.176
**Apr**	36.0	34.7	56.3	-	0.690	0.403	0.325	0.294	0.264	0.291	0.140	0.176
**May**	39.8	35	48.2	69.0	-	0.509	0.314	0.267	0.248	0.262	0.150	0.144
**Jun**	43.3	36.7	34.0	40.3	50.9	-	0.367	0.382	0.331	0.349	0.161	0.186
**Jul**	29.8	31.3	31.9	32.5	31.4	36.7	-	0.584	0.567	0.580	0.368	0.353
**Aug**	35.6	39.0	40.1	29.4	26.7	38.2	58.4	-	0.573	0.547	0.259	0.277
**Sep**	29.8	30.9	35.3	26.4	24.8	33.1	56.7	57.3	-	0.894	0.446	0.368
**Oct**	30.9	31.8	36.5	29.1	26.2	34.9	58.0	54.7	89.4	-	0.422	0.428
**Nov**	23.0	20.3	16.1	14.0	15.0	16.1	36.8	25.9	44.6	42.2	-	0.698
**Dec**	27.3	21.5	17.6	17.6	14.4	18.6	35.3	27.7	36.8	42.8	69.8	-

### Effect of elevation-month interaction on leaf beetle communities in Sierra de San Carlos

**Figure 11.**
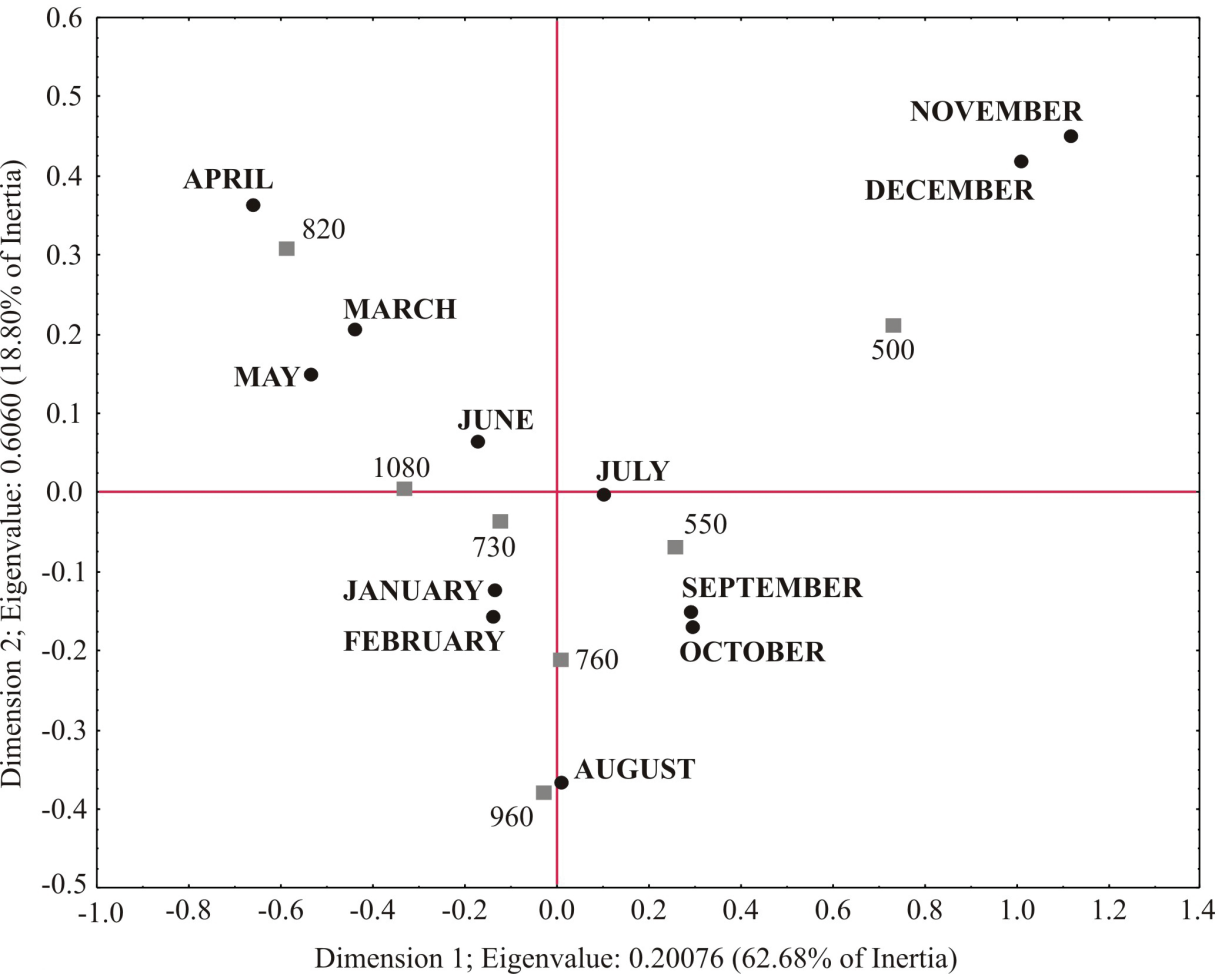
Correspondence analysis of chrysomelid abundance obtained per month at each elevational site in Sierra de San Carlos, Mexico.

The Correspondence analysis showed no significant associations in the number of species obtained by site for each month (Total Inertia=0.08796, Chi²=74.147, *df*=66, *p*=0.23014). However, the association of abundance between sites and months was significant (Total Inertia=0.32237, Chi²=993.24, *df*=66, *p*=0.0000). The most clear associations were observed between abundance obtained at lower elevations (500 and 550 masl) and the period comprised by September to December, while the number of specimens in Site 3 (960 masl) were highly related to August. January and February were principally associated with Site 5 (730 masl) and Site 2 (760 masl). The March-May period was associated with Site 6 (820 masl), and the abundance found at the highest elevation site (1080 masl) was predominantly related with June (Figure 11).

## Discussion

### Faunistic inventory and biodiversity of Chrysomelidae in Sierra de San Carlos

The 113 species of Chrysomelidae recorded in this study document that the Sierra de San Carlos represents a proportion close to 50% of the total leaf beetle species richness presently reported from Tamaulipas (Niño-Maldonado et al. 2014a) and 5% of the total reported Mexican leaf beetle fauna (Ordóñez-Reséndiz et al. 2014). Also, *Trichaltica scabricula* represents a new species record for the chrysomelid fauna of Mexico, since this species was previously known only from numerous states in the United States, including Texas (Riley et al. 2003). Additionally, the high proportion of individuals identified as unnamed morphospecies in this and other studies suggests that the actual species richness of Chrysomelidae from Tamaulipas is still greater. The number of species recorded here from Sierra de San Carlos is lower when compared to other similar studies conducted with similar methods in the region. The most related study was done in Peregrina Canyon, approximately 100 km to the southwest, near Ciudad Victoria also in the state of Tamaulipas, from which 240 total samples, 2,228 specimens and 157 species were obtained (Sánchez-Reyes et al. 2014). In Sierra de San Carlos, 1,008 samples and 3,200 specimens were obtained, but only 109 species were recorded. Still, even with the low number of species obtained, Galerucinae was the most dominant subfamily, which is consistent with the observed patterns in various other studies (Bouzan et al. 2015), including those conducted in other areas of Tamaulipas and in other states in Mexico (Niño-Maldonado et al. 2005, Ordóñez-Reséndiz and López-Pérez 2009, Sánchez-Reyes et al. 2014).

Although the sampled area for both studies was almost the same (33,600 m^2^ in Sierra de San Carlos *vs.* 37,500 m^2^ in Peregrina Canyon), the total and site-season inventory completeness in Peregrina Canyon was close to 70% (Sánchez-Reyes et al. 2014), suggesting a higher proportion of species to be added to that inventory (Jiménez-Valverde and Hortal 2003), while values for this study were close to or above 90%. Moreover, seven different vegetational communities were sampled in this study, while only three sites were studied in Peregrina Canyon. These data indicate that the number of chrysomelid species present in Sierra de San Carlos was considerably less than the sites within the Sierra Madre Oriental. Because leaf beetles are dependent of their associated vegetational communities, we expected to find a higher species richness at our study area. Our data suggest that the insular nature of the geographical location of Sierra de San Carlos (Arriaga et al. 2000) results in lower species richness of Chrysomelidae when compared with habitats connected to the Sierra Madre Oriental, as is the Peregrina Canyon. Also, the lesser richness of plants in Sierra de San Carlos (441 compared to 676 species, Martínez 1998, Briones-Villarreal 1991) is probably directly correlated with the smaller number of leaf beetles, when compared with Peregrina Canyon in Altas Cumbres Natural Protected Area, where at least 1,164 species of vascular plants have been documented (García-Morales et al. 2014). Contrary to species richness, the higher diversity values obtained in Sierra de San Carlos possibly reflect a lower degree of anthropogenic disturbance and a higher quality of natural resources and vegetational communities (Arriaga et al. 2000). These factors may favor a more balanced ecological process, leading to higher evenness in abundance of species and thus the higher diversity values (Magurran 2004).

### Elevational and seasonal effects on diversity patterns of Chrysomelidae

Elevation is one of the most important factors driving ecological communities, because the abiotic factors and biotic variables together modify species richness and composition of assemblages. Recent evidence suggests that the most common elevational pattern is the increase of diversity and species richness at intermediate elevations (Rahbek 2005, McCain and Grytnes 2010, Sanders and Rahbek 2012, Guo et al. 2013), which has been documented for various groups of Coleoptera (Escobar et al. 2005, Fernández et al. 2010), including Chrysomelidae (Furth 2009, Sánchez-Reyes et al. 2014). Other studies have shown a decrease of species richness with increasing elevation for various groups of insects (Wolda 1987, McCoy 1990, Sánchez-Ramos et al. 1993, Araújo and Fernandes 2003, Maveety et al. 2011, Jones et al. 2012). Indeed, it has been observed that species richness, abundance and diversity of Lepidoptera decreases with increasing elevation in Cerro El Diente (Meléndez-Jaramillo et al. 2015), which is one of the sampling localities in our study. However, the leaf beetle communities here analyzed did not show any consistent correlation with the elevation, such as those observed in other studies of Chrysomelidae along elevational gradients (Furth 2009, Sánchez-Reyes et al. 2014, Bouzan et al. 2015). It has been determined that small elevational ranges, even with more intensive sampling (as was done in Sierra de San Carlos), could exhibit non-unimodal patterns, due to a limited geographical range over which such patterns could be expressed (Guo et al. 2013). For example, the locality of Cerro El Diente includes an elevation range from 400 to 1200 masl, but in our study only four sites were sampled at this area. It is possible that a more stepped sampling design might result in consistent patterns of species richness, abundance and diversity, as observed in other studies conducted exclusively at this locality (Meléndez-Jaramillo et al. 2015, Sánchez-Reyes et al. 2015a). However, we suggest that the insular nature of Sierra de San Carlos, its geomorphic properties, and the lack of connectivity between elevational sampling sites in different localities, could be the main drivers of results here obtained, because these factors have been recognized as key determinants of biodiversity patterns within elevational gradients (Bertuzzo et al. 2016).

Although a consistent elevational pattern was not found, the high proportion of inventory completeness through all methods employed indicates that the faunistic composition obtained at each site is representative; so, the values of abundance and diversity were reliable (Jiménez-Valverde and Hortal 2003). On this basis, we affirm that 1) highest values of diversity from Sierra de San Carlos were present at the highest and lowest elevations at the Cerro El Diente locality (Site 4, Cloud forest at 1080 masl; and Site 1, Submountain scrub at 550 masl), and also 2) the highest species richness was recorded in the highest elevation site from the same locality (Site 4, 1080 masl). This could be due to the vegetational composition and the characteristics at that area, since the contrasting more humid areas in the Cloud forest in the highest elevation site must be favoring the higher values obtained of diversity and species richness. Besides, we found higher values of species richness and diversity at sites within Cerro El Diente, compared with other sites from the Ejido Carricitos y Tinajas and San Nicolás localities. Land area is a determining factor in shaping communities within elevation gradients (Körner 2007). However, this factor might not affect species richness and diversity in our study, as fewer species were collected from more extensive sampling areas, such as Site 7 and both sites from the Ejido Carricitos y Tinajas locality; contrarily, intermediate vegetational communities from Cerro El Diente, even with sampling plots distributed randomly within corresponding elevational intervals, covered a smaller area (Figures 2, 3) but presented higher values of species richness and diversity. Considering that chrysomelid communities are directly influenced by plant-associated variables (Bach 1981, Řehounek 2002, Aslan and Ayvaz 2009, Sen and Gök 2009), we attribute these results to the higher quality of the vegetational communities at the Cerro El Diente locality (Arriaga et al. 2000, CONABIO 2007). These findings highlight the significance of this locality within Sierra de San Carlos, and constitute support for its designation as an area with extreme priority for conservation. Also, the presence of Cloud forest in Sierra de San Carlos and the associated communities of leaf beetles are remarkable, since this ecosystem has a relict distribution in Mexico and is considered as an important center for high levels of biodiversity and endemism (Gual-Díaz and Rendón-Correa 2014); thus, our results contribute to the categorization of Sierra de San Carlos as a sky island and emphasize its protection urgency.

When analyzing species composition and beta diversity between sites, we observed that Sites 2 and 3 were the only sites with the same composition and a high faunistic similarity, while almost all other comparisons were different, which is contrary to other findings that show a high similarity of Lepidoptera between elevation and seasons at Cerro El Diente (Meléndez-Jaramillo et al. 2015). It has been determined that habitat heterogeneity and diverse characteristics of conservation areas can promote high beta diversity or low similarity between sites, regardless of the distance (Linzmeier and Ribeiro-Costa 2009). This was observed with the lowest site of Cerro El Diente and the other three sites within the same locality, as they formed different faunistic groups, although they are geographically close to each other. Moreover, we found that the lowest elevational sites from Sierra de San Carlos (Site 1 and Site 7) formed a faunistic group even when these were from distant localities. Undoubtedly, this is due to similar vegetational communities, which lead to similar leaf beetle faunas. Conversely, both sites were very different in terms of abundance of species, thus reflecting the specific responses of each species to abiotic and biotic characteristics at each site. This evidence suggests that differences in microhabitats result in very different assemblages of species, owing to an almost entirely different plant composition and also to the climatic or abiotic variation (Ødegaard 2006).

Regarding seasonal analysis, patterns observed at Sierra de San Carlos were different from those recorded in the most related study in Peregrina Canyon, Tamaulipas (Sánchez-Reyes et al. 2014), as our highest values of abundance, species richness and diversity were recorded in the rainy season. These findings are supported by other studies, because the dominance and increased abundance of adult leaf beetles in rainy or wet seasons is the most common result in studies of seasonal variation of this taxon and other insects (Ødegaard 2006, Furth 2009, Bouzan et al. 2015), which is highly related to the increase in plant density during this period. Besides, considering that higher seasonal peaks of abundance are associated with more marked seasons (Wolda et al. 1998), we suggest that the more seasonally dry environmental conditions at Sierra de San Carlos could be a primary factor in the species of Chrysomelidae being absent from samples or less active in the dry season, due to reduction in quality and availability of host plants, and to abiotic conditions (Medeiros and Vasconcellos-Neto 1994, Awmack and Leather 2002, Ishihara and Ohgushi 2006). Furthermore, evidence has shown that phytophagous insects locate temporary refugia when environmental conditions are less suitable (Janzen 1973), thus not being sampled in sweep catches and resulting in low values of abundance, species richness and diversity during dry seasons in non-refugia sites.

According to cluster analysis, three groups were formed, based on faunistic similarity between months: November and December, January to June, and July to October. This inconsistency between the four climate seasons (dry/rainy) and the clustering of months by species compositions is possibly due to differential species responses to seasonal variations, as their temporal niche requirements are very distinct (Linzmeier and Ribeiro-Costa 2013). Similarly, seasonal variations, monthly composition and similarity patterns in leaf beetles reflect different ecological and biological strategies of each species, since reproduction and adult feeding phases may be different at certain times of the year. In Chrysomelidae, this has been attributed to abiotic variation during seasons, such the photoperiod, temperature, relative humidity, and to the quality and availability of host plants (Linzmeier and Ribeiro-Costa 2008, 2013). Also, seasonal and monthly patterns could reflect an existing sequence between Chrysomelidae and other phytophagous species to avoid competition and allow optimum resource exploitation (Pérez-Barroeta and Gurrea-Sanz 1994).

In addition to the influence of the geographical location of Sierra de San Carlos, the responses of the chrysomelid community in this study, being elevational, seasonal or both, are suggested to be driven by host plants and vegetational associated variables (Bach 1981, Erelli et al. 1998, Řehounek 2002, Aslan and Ayvaz 2009, Sen and Gök 2009). So, although not investigated here, the study of relationships between Chrysomelidae and their associated vegetational communities is very important to understand the distribution patterns of this taxon. However, it has been noted that the interplay between the biotic and abiotic environment shapes consumer diversity along elevational gradients (Sundqvist et al. 2013). Therefore, changes in abundance, species richness and diversity within each locality, site and season/month could be driven by other factors related to elevation change, such as climate variables or abiotic environment (temperature, humidity, Körner 2007), which could be influencing the activity patterns of Chrysomelidae and the capacity of each species to obtain resources (Flinte et al. 2011, Bouzan et al. 2015). This influence of climate has been demonstrated in this study, since precipitation and temperature were significantly correlated with species richness; other studies on Chrysomelidae have reported similar effects (Linzmeier and Ribeiro-Costa 2008, Sánchez-Reyes et al. 2014).

Besides, the results obtained through Correspondence analysis confirm the same tendency of an interaction between abiotic and biotic factors on distribution of Chrysomelidae, because they show that abundance of the leaf beetle community at each site is associated with specific months. This was observed, for example, with the significant association of the lowest sites and abundance obtained at the rainy months, or that observed between Site 3 (960 masl) and August. Consequently, these associations suggest unique and specific temporal-site conditions for the communities of Chrysomelidae, which surely are the result of monthly changes in both environmental (abiotic) conditions and plant variables along the elevational gradient. Since elevational and temporal responses of biological communities arise from the effects (direct or indirect) of these gradients on each species (Hodkinson 2005, Sundqvist et al. 2013, Guo et al. 2013), future research on Chrysomelidae in Mexico needs to be targeted at the specific species patterns and their relationships with environmental variation, as well as at specific interactions between leaf beetle species and other ecosystem components.

## Conclusions

The species richness of Chrysomelidae in Sierra de San Carlos was not as high as expected for an area with extreme priority for conservation, which could be the result of the geographical position of the study area. However, the high quality of the vegetational communities is presumably associated to the high diversity values. This is true for the highest site of our elevational gradient, where the highest values of species richness and diversity were obtained, and which must surely be associated with the environmental conditions of the cloud forest vegetation at that site, thus emphasizing the conservation urgency of this relict area and supporting the presence of a sky island within Sierra de San Carlos; hence, its high importance for biological conservation and for investigations of leaf beetle distribution.

The first record of a species for Mexico in Sierra de San Carlos is remarkable. Moreover, many of the specimens here determined as morphospecies could be later recognized as new distribution records, or new species. So, it is possible that leaf beetle species at Sierra de San Carlos constitute a very distinctive faunistic assemblage from other chrysomelid faunas in Mexico, which, added to the absence of a clear elevational pattern, suggests a strong effect of the insular geographical position and other geomorphic characteristics of Sierra de San Carlos on the Chrysomelidae distribution. Rainy season was associated with higher values of the ecological parameters of Chrysomelidae, being consistent with general patterns of temporal distribution of leaf beetles.

Regarding leaf beetle composition, we found evidence that different microhabitats, regardless of the distance, as well as different months, support distinct faunistic assemblages. Most importantly, communities within these particular sites are differentially influenced by changing conditions during seasonal/month variation, as suggested by the Correspondence analysis, and by the direct correlation of temperature and precipitation with species richness. These differences and variations in faunistic composition within the elevational and temporal gradients surely mirror differences in floristic composition and abiotic variables, since both are related to leaf beetle distribution. However, these changes must be addressed at a specific level, because the niche requirements of each species are very distinct. Since this is one of few studies conducted in Mexico concerning chrysomelid biodiversity and the variation along natural gradients, it is important that future research accounts for the specific influence of environmental modification (biotic and abiotic) on chrysomelid species at Sierra de San Carlos and other ecological gradients within Mexico. Also, forthcoming studies must address biogeographical relationships of chrysomelid species existent within this and others areas in the country.

## Appendix 1

**Table A.** Taxonomic checklist and abundance of Chrysomelidae by site and month in Sierra de San Carlos, Mexico. Site column: numbers in square brackets refer to the plot number where the species was collected within that site; see Material and methods (Tables 1, 2, 3 and Figures 2–5) for detailed data (coordinates, elevation, type of vegetation, spatial location) of each site-plot. Month column: numbers in parenthesis refer to the total abundance obtained for that month. Marked (*) species were obtained only by collecting, independent of the standardized sweeps. NR=New record for Mexico.

Taxon	Site [plot]	Month (abundance)
CRIOCERINAE Latreille, 1807		
Tribe Lemini Heinze, 1962		
*Lema balteata* LeConte, 1884	Site 4 [4]	Aug (1)
*Lema opulenta* Harold, 1874	Site 5 [6]	Aug (1)
*Lema* sp. 1	Site 3 [2]	Nov (1), Dec (1)
*Neolema* sp. 1	Site 1 [9]	Aug (1)
	Site 2 [4]	Sep (1), Oct (1)
	Site 3 [2]	Jul (1), Aug (1)
	Site 4 [4, 7, 8, 9, 10]	Jun (1), Jul (1), Aug (5), Sep (1), Oct (1)
*Neolema* sp. 2	Site 5 [1]	May (1)
*Oulema* sp. 1	Site 3 [3, 11]	Jul (1), Sep (1), Oct (1)
	Site 5 [3]	Aug (1)
*Oulema* sp. 2	Site 3 [1, 12]	Aug (1), Sep (1), Oct (1)
*Oulema* sp. 3	Site 4 [1, 7]	Jul (1), Aug (1)
CASSIDINAE Gyllenhal, 1813		
Tribe Chalepini Weise, 1910		
*Brachycoryna pumila* Guérin-Méneville, 1844	Site 1 [1, 2, 4, 7, 9, 11]	May (2), Jun (1), Jul (1), Sep (3), Oct (2)
	Site 2 [1, 2, 3, 6, 7, 9, 11]	Jan (2), Feb (2), Apr (2), Jun (1), Jul (3), Aug (1), Sep (2), Oct (2)
	Site 4 [3, 4, 9]	May (1), Jul (1), Sep (1), Oct (1), Nov (1), Dec (1)
	Site 6 [7, 8, 11, 12]	Jan (1), Feb (2), May (1), Jun (2), Jul (1), Aug (4)
	Site 7 [1, 5]	Jul (1), Aug (1)
*Chalepus verticalis* (Chapuis, 1877)	Site 7 [6]	Sep (1), Oct (1)
*Sumitrosis inaequalis* (Weber, 1801)	Site 1 [6, 7, 8]	Jan (1), Feb (1), Jul (1), Sep (1), Oct (1), Nov (2)
	Site 2 [5, 8, 9, 10, 11, 12]	Jan (1), Feb (3), Mar (2), May (1), Jun (2), Jul (3), Aug (1), Sep (2), Oct (1), Nov (6), Dec (2)
	Site 3 [3, 6, 8]	Jun (1), Jul (1), Sep (1), Oct (1)
	Site 4 [5, 6, 7, 8, 9, 10, 11, 12]	Jan (1), Feb (1), May (6), Jun (2), Jul (8), Aug (4), Sep (7), Oct (5)
	Site 5 [2, 8, 11]	Jul (1), Aug (1), Sep (1), Oct (1)
	Site 6 [1, 3, 9]	May (1), Jul (4)
Tribe Mesomphaliini Hope, 1840		
*Ogdoecosta juvenca* (Boheman, 1854)	Site 4 [8, 9]	Jun (3), Jul (2), Aug (1)
Tribe Ischyrosonychini Chapuis, 1875		
*Physonota alutacea* Boheman, 1854	Site 1 [4]	Sep (1), Oct (1)
	Site 7 [1, 2, 12]	Jun (1), Sep (7), Oct (7), Nov (3), Dec (2)
Tribe Cassidini Gyllenhal, 1813		
Coptocycla (Psalidonota) texana (Schaeffer, 1933)*	Site 1	May
*Helocassis clavata* (Fabricius, 1798)	Site 2 [5]	May (1), Jul (1)
	Site 3 [4]	May (1)
	Site 5 [3]	Jun (1)
*Helocassis crucipennis* (Boheman, 1855)	Site 5 [7]	Aug (1)
	Site 6 [1]	Jul (1)
*Metrionella bilimeki* Spaeth, 1932	Site 4 [8, 9, 10, 11]	May (1), Jul (3), Aug (3), Sep (4), Oct (3)
CHRYSOMELINAE Latreille, 1802		
Tribe Chrysomelini Latreille, 1802		
Subtribe Doryphorina Motschulsky, 1860		
*Labidomera suturella* Chevrolat, 1844	Site 3 [3]	Nov (1), Dec (1)
	Site 4 [11]	Jul (1)
Subtribe Chrysomelina Latreille, 1802		
*Plagiodera semivittata* Stål, 1860	Site 2 [5, 10, 12]	Jan (1), Feb (1), Jun (2), Sep (2), Oct (2), Nov (1), Dec (1)
	Site 3 [3, 5, 7, 8]	Jan (1), Feb (1), May (2), Jun (6)
	Site 4 [2, 3]	Apr (1), Sep (1), Oct (1)
*Plagiodera thymaloides* Stål, 1860	Site 2 [8, 10, 11]	Jun (2), Jul (1), Aug (2)
	Site 3 [3, 8]	Jun (2)
GALERUCINAE Latreille, 1802		
Tribe Galerucini Latreille, 1802		
Group Coelomerites Chapuis, 1875		
*Coraia subcyanescens* (Schaeffer, 1906)	Site 1 [1]	Jun (1)
*Miraces aeneipennis* Jacoby, 1888	Site 1 [4, 10, 11]	Aug (1), Sep (2), Oct (2), Nov (2), Dec (2)
	Site 2 [3, 4, 7]	Sep (6), Oct (4)
	Site 3 [4, 9, 10, 12]	Jun (1), Jul (1), Sep (5), Oct (5)
	Site 7 [12]	Jul (1), Nov (1), Dec (1)
Group Schematizites Chapuis, 1875		
*Monoxia* sp. 1	Site 1 [4]	Sep (1), Oct (1)
*Ophraella* sp. 1	Site 1 [9]	Jan (1), Feb (1)
Tribe Metacyclini Chapuis, 1875		
*Malacorhinus acaciae* (Schaeffer, 1906)	Site 7 [1, 6, 10]	Jun (3), Sep (3), Oct (3)
*Malacorhinus* sp. 1	Site 4 [4]	Nov (1), Dec (1)
Tribe Luperini Chapuis, 1875		
Subtribe Diabroticina Chapuis, 1875		
Group Diabroticites Chapuis, 1875		
*Acalymma invenustum* Munroe & Smith, 1980	Site 1 [5]	Sep (1), Oct (1)
	Site 3 [5]	Sep (1), Oct (1)
	Site 4 [4]	Aug (2)
*Gynandrobrotica lepida* (Say, 1835)	Site 5 [1, 2, 6, 7, 8, 10, 11, 12]	Apr (1), Aug (3), Sep (11), Oct (9), Nov (6), Dec (5)
	Site 6 [1, 3, 4, 6, 10, 11, 12]	Jul (1), Aug (2), Sep (12), Oct (9), Nov (3), Dec (2)
Group Cerotomites Chapuis, 1875		
*Cyclotrypema furcata* (Olivier, 1808)	Site 4 [6, 10, 12]	Jun (2), Aug (2)
	Site 5 [6]	Aug (1)
Tribe Alticini Newman, 1835		
*Acallepitrix* sp. 1	Site 1 [12]	Sep (1), Oct (1)
	Site 2 [3, 8]	Jan (2), Feb (2), Nov (1)
	Site 3 [9]	Mar (1)
	Site 4 [7, 8]	Mar (1), Aug (1)
	Site 5 [1, 2, 3, 6, 12]	Jan (1), Feb (1), Mar (1), Aug (3), Sep (5), Oct (3), Nov (2), Dec (2)
	Site 6 [1, 2, 4, 5, 6, 8, 9]	Jan (3), Feb (8), Mar (11), Apr (1), Jul (2), Aug (2)
*Acallepitrix* sp. 2	Site 5 [3, 7]	Aug (2)
	Site 6 [1, 2]	Mar (3), Apr (1), May (1), Jul (1)
*Acallepitrix* sp. 3	Site 4 [4]	Jun (1)
*Acallepitrix* sp. 4	Site 1 [2]	Sep (1), Oct (1)
	Site 3 [2, 3, 11]	Jul (2), Aug (1), Sep (2), Oct (2)
	Site 4 [2, 4, 6, 7, 8, 9, 11, 12]	Jun (1), Jul (3), Aug (4), Sep (4), Oct (2)
	Site 5 [3]	Sep (1), Oct (1)
	Site 6 [8]	Jun (2), Sep (1), Oct (1)
*Acrocyum dorsalis* Jacoby, 1885	Site 2 [3, 10]	Mar (1), Sep (1), Oct (1)
	Site 3 [2]	Jan (1), Feb (1)
*Alagoasa decemguttata* (Fabricius, 1801)	Site 4 [6, 8]	Apr (1), Sep (1), Oct (1)
*Altica* sp. 1	Site 4 [10]	Jul (1)
*Altica* sp. 2	Site 3 [1, 10]	Jul (1), Aug (1)
*Altica* sp. 3	Site 5 [11]	Apr (1)
*Blepharida rhois* (Forster, 1771)	Site 7 [1, 4, 11, 12]	Aug (1), Sep (2), Oct (2), Nov (3), Dec (2)
*Centralaphthona diversa* (Baly, 1877)	Site 1 [2, 3, 6, 12]	Jan (2), Feb (3), Jun (2), Aug (1), Sep (1), Oct (1)
	Site 2 [2, 4, 10, 12]	Jan (2), Feb (2), Mar (1), Jun (7), Aug (6), Sep (5), Oct (4), Nov (2), Dec (2)
	Site 3 [1, 2, 3, 4, 5, 6, 7, 8, 9, 10, 11, 12]	Jan (16), Feb (29), Mar (4), May (4), Jul (4), Aug (15), Sep (3), Oct (2)
	Site 4 [1, 2, 3, 4, 6, 8, 9, 10, 11, 12]	Jan (4), Feb (8), Mar (19), Apr (4), May (2), Jun (4), Jul (3), Aug (5), Sep (2), Oct (2)
	Site 5 [1, 2, 3, 4, 6, 11]	Jan (2), Feb (2), Apr (1), May (1), Jun (3), Jul (2), Aug (3), Sep (1), Oct (1)
	Site 6 [1, 4, 5, 6, 7, 8, 9, 10, 11, 12]	Jan (8), Feb (17), Mar (3), Apr (1), May (2), Jun (10), Jul (1), Aug (9), Sep (2), Oct (2)
	Site 7 [1, 3]	Apr (1), Aug (1)
*Centralaphthona* sp. 2	Site 2 [3, 4, 11]	Jan (1), Feb (1), Jul (1), Aug (1)
	Site 3 [9]	May (1)
	Site 6 [9]	May (1)
*Centralaphthona* sp. 3	Site 3 [3, 7]	Jan (1), Feb (1), Aug (1)
	Site 4 [9, 10]	Jan (1), Feb (1), Mar (1)
	Site 5 [9, 11, 12]	Jun (1), Aug (2)
	Site 6 [3, 4]	May (1), Jul (1)
*Centralaphthona* sp. 4	Site 6 [11]	Jul (1)
*Chaetocnema* sp. 1	Site 1 [2, 3, 7]	Jun (1), Jul (18), Aug (1), Sep (10), Oct (8)
	Site 5 [2, 3, 4]	Aug (3), Sep (3), Oct (3)
	Site 6 [6, 10, 11]	Aug (1), Sep (4), Oct (3)
*Chaetocnema* sp. 2	Site 1 [11]	Nov (1), Dec (1)
	Site 2 [10]	Nov (1), Dec (1)
	Site 3 [3, 8]	Apr (1), Nov (1), Dec (1)
	Site 4 [5, 10, 11, 12]	Jan (2), Feb (3), Mar (1), Aug (7)
	Site 5 [2, 11]	Jan (1), Feb (1), Mar (1)
	Site 6 [6, 9]	Jan (1), Feb (1), Apr (2)
*Chaetocnema* sp. 3	Site 1 [6]	Sep (1), Oct (1)
	Site 2 [3]	Jan (1), Feb (1)
	Site 3 [9]	May (1)
	Site 4 [1, 12]	May (3)
	Site 5 [2, 4, 5, 6]	Jan (1), Feb (1), Apr (1), May (5), Sep (1), Oct (1)
	Site 6 [6, 11]	Jan (2), Feb (2), Jun (1)
	Site 7 [1, 3, 6]	Nov (3), Dec (3)
*Chaetocnema* sp. 4	Site 6 [11]	May (1)
*Chrysogramma* sp. 1	Site 1 [6]	Apr (1)
	Site 2 [1, 2, 3, 4, 6, 7, 9, 10, 11, 12]	Jan (12), Feb (41), Mar (45), Apr (6), May (2), Jul (1)
	Site 3 [1, 2, 3, 4, 5, 6, 7, 8, 10, 11, 12]	Jan (14), Feb (34), Mar (26), Apr (8), May (3)
*Dibolia* sp. 1	Site 4 [4]	Aug (1)
*Disonycha glabrata* (Fabricius, 1781)	Site 5 [1, 6]	Aug (2)
*Disonycha stenosticha* Schaeffer, 1931	Site 1 [6]	Nov (2)
	Site 2 [3, 4, 6, 8, 11, 12]	Jul (2), Sep (2), Oct (2), Nov (5), Dec (1)
	Site 3 [1, 7, 9]	Jul (1), Nov (3), Dec (2)
	Site 6 [8]	Aug (1)
*Disonycha teapensis* Blake, 1933	Site 3 [1]	Sep (1), Oct (1)
*Dysphenges* sp. 1	Site 1 [4, 10]	Jul (2)
	Site 3 [1, 5, 8, 9]	Jul (4), Sep (1), Oct (1)
	Site 7 [2, 9]	Jul (1), Sep (1)
*Epitrix* sp. 1	Site 3 [8]	Jan (1), Feb (1)
	Site 4 [3, 5, 6, 8, 9, 10]	Jan (1), Feb (1), Mar (2), May (3), Jun (2), Aug (1)
	Site 5 [1, 2, 3, 4, 6, 7, 8, 12]	Jan (2), Feb (2), Mar (1), May (1), Jun (2), Jul (1), Aug (2), Sep (1), Oct (1)
	Site 6 [6, 9, 11, 12]	Jan (2), Feb (2), May (1), Aug (1), Sep (3), Oct (1)
*Epitrix* sp. 2	Site 1 [6]	Nov (1)
	Site 2 [2, 10]	Jan (1), Feb (1), Mar (1), Nov (1), Dec (1)
	Site 3 [1, 5, 8, 10, 11, 12]	Jan (3), Feb (3), Mar (4), Jul (1), Aug (1)
	Site 4 [5, 7, 11, 12]	Jan (1), Feb (1), Mar (2), Apr (1)
	Site 5 [6, 8]	Jan (1), Feb (1), Jun (1)
*Epitrix* sp. 3	Site 2 [9]	Mar (1)
	Site 4 [1, 9, 12]	Jan (1), Feb (1), Mar (2)
	Site 7 [6]	Sep (1), Oct (1)
*Epitrix* sp. 4	Site 5 [3]	Jun (2), Aug (2)
*Hypolampsis* sp. 1	Site 4 [6]	Jun (1)
*Kuschelina laeta* (Perbosc, 1839)	Site 7 [6]	Aug (1)
*Longitarsus* sp. 1	Site 1 [6]	Mar (1)
	Site 2 [2]	Jul (2)
	Site 4 [1, 3]	Mar (1), Apr (1)
	Site 7 [1, 6]	Jan (1), Feb (1), Sep (1), Oct (1)
*Longitarsus* sp. 2	Site 1 [8]	May (1)
	Site 2 [2, 5]	Jun (1), Aug (1)
	Site 3 [3, 4, 5, 8, 10]	Jul (1), Aug (1), Nov (3), Dec (2)
	Site 4 [1, 3, 4, 5, 7, 8, 9, 10]	Jan (3), Feb (4), Mar (5), Apr (1), May (1), Jun (1), Jul (5), Aug (2), Sep (3), Oct (1)
	Site 5 [1, 3, 5, 8]	May (1), Aug (3)
	Site 6 [5, 10]	Jul (2), Aug (1)
	Site 7 [1]	Jul (1)
*Lupraea* sp. 1	Site 3 [5]	Sep (1), Oct (1)
	Site 4 [1, 4, 5, 7. 8, 10, 11]	Apr (3), Jul (3), Aug (7), Sep (2), Oct (1)
*Lupraea* sp. 2	Site 3 [4]	Sep (1), Oct (1)
	Site 4 [1, 4]	Sep (4), Oct (2)
*Lysathia* sp. 1	Site 5 [6]	Mar (1)
*Margaridisa atriventris* (Melsheimer, 1847)	Site 2 [2, 5, 12]	Aug (3)
	Site 3 [1, 4, 5, 6, 8, 10, 11, 12]	Jan (1), Feb (1), May (1), Jun (1), Jul (7), Aug (14), Sep (2), Oct (2)
	Site 4 [8, 9]	Mar (1), Jul (2)
	Site 5 [1, 3, 10, 11]	Mar (1), Jun (1), Jul (2), Aug (2), Sep (1), Oct (1)
	Site 6 [1, 2, 4, 5, 7, 12]	Jan (1), Feb (1), May (1), Jun (2), Jul (18), Aug (5)
	Site 7 [6]	Aug (1)
*Monomacra* sp. 1	Site 4 [7]	May (2)
*Parchicola* sp. 1	Site 2 [2, 5, 7]	Jul (3), Aug (1), Sep (1), Oct (1)
	Site 3 [1, 10, 12]	Jul (4)
*Phydanis nigriventris* Jacoby, 1891	Site 3 [3]	Aug (1)
	Site 7 [11]	Aug (1)
*Phyllotreta* sp. 1	Site 1 [5, 9]	Mar (2)
	Site 2 [7]	Jan (1), Feb (1)
	Site 3 [2, 11, 12]	Mar (3), May (1)
	Site 4 [7, 9]	Mar (4), May (1)
	Site 5 [1]	Apr (1)
	Site 6 [6, 9, 10]	Mar (15)
*Plectrotetra* sp. 1	Site 3 [3]	Mar (1)
	Site 4 [1, 2, 3, 4, 5, 10, 12]	Apr (7), May (6), Jun (7)
*Sphaeronychus fulvus* (Baly, 1879)	Site 4 [4, 5, 9, 10, 11]	Jan (1), Feb (2), Mar (8), Jul (1), Sep (2), Oct (2)
*Syphrea* sp. 1	Site 2 [1]	Aug (2)
	Site 5 [2]	Jul (1)
	Site 7 [1, 5, 6, 7, 8, 11]	Mar (1), Apr (1), Jun (1), Jul (6), Aug (7), Sep (1), Oct (1), Nov (1), Dec (1)
*Syphrea* sp. 2	Site 1 [8]	Aug (1)
	Site 2 [2, 3, 5, 6, 8, 9, 10, 11, 12]	Jan (2), Feb (2), May (1), Jun (2), Jul (3), Aug (42), Sep (30), Oct (24)
	Site 3 [1, 2, 3, 4, 5, 6, 7, 8, 9, 10, 11, 12]	May (6), Jun (3), Jul (14), Aug (87), Sep (43), Oct (31)
	Site 4 [1, 2, 3, 4, 5, 6, 8, 9, 10, 11, 12]	Jan (3), Feb (5), Mar (4), Apr (3), May (1), Jun (1), Jul (12), Aug (6), Sep (2), Oct (2)
	Site 5 [5, 6, 8, 11]	Mar (1), May (3), Aug (2)
	Site 6 [1, 2, 3, 4, 5, 8, 12]	Jan (10), Feb (19), Mar (47), Apr (34), May (6), Jun (4), Jul (6), Aug (3), Sep (3), Oct (3), Nov (2), Dec (2)
*Systena* sp. 1	Site 7 [7]	Jul (1)
*Trichaltica scabricula* (Crotch, 1873). NR	Site 7 [6, 11, 12]	May (4)
EUMOLPINAE Hope, 1840		
Tribe Eumolpini Hope, 1840		
Group Iphimeites Chapuis, 1874		
*Brachypnoea* sp. 1	Site 2 [3,4]	Mar (2)
	Site 3 [4, 6, 8, 9, 10]	Mar (3), Apr (8), Sep (4), Oct (3)
	Site 4 [1, 2, 7, 11, 12]	Apr (1), May (5), Jun (3), Aug (1)
	Site 6 [3, 6, 10]	Mar (1), Apr (1), Jun (1)
*Brachypnoea* sp. 2	Site 1 [1]	Jun (1)
*Colaspis melancholica* Jacoby, 1881	Site 2 [9]	Jun (1), Jul (1)
	Site 4 [4]	Jun (1)
*Deuteronoda suturalis* (Lefèvre, 1878)*	Site 5	Jun
Tribe Adoxini Baly, 1863		
Group Leprotites Chapuis, 1874		
*Xanthonia* sp. 1	Site 2 [3]	Feb (1)
	Site 3 [5, 6, 7, 8, 9, 12]	Apr (2), May (1), Jun (3), Jul (2)
	Site 4 [1, 2, 3, 4, 5, 6, 7, 9, 10, 11, 12]	Apr (5), May (7), Jun (6), Jul (5), Aug (2)
	Site 5 [2, 4, 5, 6, 8, 9, 10, 11, 12]	Mar (12), Apr (13), May (22), Jun (4), Jul (1), Aug (2)
	Site 6 [1, 2, 3, 4, 5, 6, 7, 8, 9, 10, 11, 12]	Jan (6), Feb (12), Mar (56), Apr (59), May (37), Jun (10), Aug (8)
*Xanthonia* sp. 2	Site 4 [4, 5, 6, 8, 9, 10, 11, 12]	Apr (12), May (7), Jun (6), Jul (1)
*Xanthonia* sp. 3	Site 3 [7, 10]	Sep (3), Oct (3)
	Site 4 [12]	Sep (1), Oct (1)
*Xanthonia* sp. 4	Site 2 [11]	Apr (1)
	Site 3 [4, 6, 7, 10]	Mar (4), Apr (2)
*Xanthonia* sp. 5	Site 3 [5, 12]	Jul (1), Aug (2), Sep (2), Oct (2)
	Site 4 [5, 8, 9, 11]	May (1), Jun (1), Jul (6)
CRYPTOCEPHALINAE Gyllenhal, 1813		
Tribe Cryptocephalini Gyllenhal, 1813		
Subtribe Pachybrachina Chapuis, 1874		
*Griburius montezuma* (Suffrian, 1852)	Site 7 [4]	Jun (2)
*Griburius* sp. 1	Site 7 [1]	Jun (2)
*Pachybrachis* sp. 1	Site 1 [1, 3, 4, 5, 9, 10, 11]	Mar (1), Apr (1), Jul (1), Aug (2), Sep (4), Oct (4), Nov (1), Dec (1)
	Site 2 [3, 4, 5, 6, 8, 11]	Apr (1), Aug (3), Sep (3), Oct (3)
	Site 4 [12]	Jul (1)
	Site 5 [2, 4, 5]	May (2), Sep (3), Oct (3)
	Site 6 [6, 7, 8, 9, 10, 12]	Mar (7), Apr (3), May (3), Jun (4), Jul (1), Aug (4), Sep (3), Oct (3)
	Site 7 [1, 2, 3, 4, 5, 6, 7, 9, 11, 12]	Jan (4), Feb (5), Mar (5), Apr (1), May (1), Jun (2), Jul (3), Aug (5), Sep (5), Oct (4), Nov (3), Dec (3)
*Pachybrachis* sp. 2	Site 2 [3, 5, 7, 9]	Mar (1), Apr (5), May (5), Aug (1)
*Pachybrachis* sp. 3	Site 5 [4]	Jul (1)
	Site 6 [12]	Jun (1)
*Pachybrachis* sp. 4	Site 1 [3, 4, 7, 10, 11, 12]	Jan (2), Feb (2), Mar (4), May (1), Aug (1)
	Site 3 [3]	May (1)
	Site 4 [2, 12]	Jun (1), Sep (1), Oct (1)
	Site 7 [4, 7, 8, 9, 11, 12]	Jan (4), Feb (14), Mar (3)
*Pachybrachis* sp. 5	Site 1 [11]	Mar (1), Aug (1)
	Site 2 [3, 4, 7, 11]	Mar (1), Jun (1), Aug (2)
	Site 3 [3, 4, 7, 8, 10]	Mar (1), Apr (3), May (1), Jun (2), Jul (7), Aug (1)
	Site 4 [1, 2, 7, 9, 12]	Apr (1), May (4), Jun (1), Jul (1)
	Site 5 [4]	Apr (1)
	Site 6 [10]	Jul (1)
	Site 7 [12]	Aug (4)
*Pachybrachis* sp. 6	Site 2 [7]	Sep (1), Oct (1)
*Pachybrachis* sp. 7	Site 1 [4]	Jun (1), Aug (1), Sep (2), Oct (2)
	Site 2 [3]	Sep (1), Oct (1)
	Site 7 [2, 3, 4, 5, 7, 8, 9, 10]	Jan (2), Feb (2), Mar (1), Jun (7), Jul (5), Aug (3), Sep (6), Oct (6)
*Pachybrachis* sp. 8	Site 1 [4, 8]	Jan (1), Feb (1), Sep (1), Oct (1)
	Site 2 [1]	Mar (1)
	Site 3 [7]	Jul (1)
	Site 7 [1, 3, 4, 6, 8, 9, 11, 12]	Jan (1), Feb (2), Mar (3), Jun (1), Jul (1), Aug (1), Sep (2), Oct (2), Nov (7), Dec (5)
*Pachybrachis* sp. 9	Site 2 [3, 4, 5, 9]	Mar (2), Apr (2), May (1), Jul (1), Aug (1)
	Site 7 [3]	Apr (1)
*Pachybrachis* sp. 10	Site 5 [1]	Jan (2), Feb (2), Aug (1)
*Pachybrachis* sp. 11	Site 5 [2]	Jan (1), Feb (1)
*Pachybrachis* sp. 12	Site 1 [4]	Sep (1), Oct (1)
Subtribe Cryptocephalina Gyllenhal, 1813		
*Cryptocephalus downiei* Riley & Gilbert, 2000	Site 7	Apr (Sánchez-Reyes et al. 2015b)
*Cryptocephalus duryi* Schaeffer, 1906*	Site 5	Apr
*Cryptocephalus guttulatus* Olivier, 1808	Site 3 [11]	Jul (1)
	Site 4 [1, 8, 9, 10, 11, 12]	Jun (1), Jul (5), Aug (2)
	Site 5 [3]	Jun (1)
*Cryptocephalus trizonatus* Suffrian, 1858	Site 1 [1, 6, 9, 11]	Mar (1), Jun (1), Sep (1), Oct (1), Nov (2)
	Site 2 [3, 10, 12]	Aug (1), Sep (1), Oct (1), Nov (1)
	Site 7 [1, 4, 9, 10, 11]	Apr (1), Aug (1), Nov (5), Dec (2)
*Cryptocephalus umbonatus* Schaeffer, 1906	Site 1 [4, 11, 12]	Jul (1), Sep (2), Oct (1)
	Site 2 [3, 5, 6, 8, 9, 10, 11]	Jun (9), Jul (1), Aug (4), Sep (1), Oct (1)
	Site 3 [3, 7, 8, 11, 12]	Jun (2), Jul (2), Sep (1), Oct (1)
	Site 4 [4]	Aug (1)
	Site 5 [1, 2, 12]	Aug (1), Sep (2), Oct (2)
	Site 7 [1]	Aug (1)
*Diachus auratus* (Fabricius, 1801)	Site 1 [5, 6, 9, 10]	Mar (4)
	Site 2 [6]	Jan (1), Feb (1)
	Site 3 [2, 10]	Mar (1), Apr (2)
	Site 4 [2, 9, 10, 12]	Jan (1), Feb (2), Mar (5), May (1), Jun (2), Sep (1), Oct (1)
	Site 5 [3]	Mar (2)
	Site 6 [1]	Jan (1), Feb (1)
	Site 7 [7]	Jan (2), Feb (3)
*Diachus* sp. 1	Site 7 [1, 2, 3, 4, 5, 6, 7, 8, 9, 10, 12]	Jan (10), Feb (15), Mar (19), Apr (8), May (4), Jun (6), Jul (55), Aug (40), Sep (69), Oct (50), Nov (98), Dec (44)
Tribe Clytrini Lacordaire, 1848		
Subtribe Clytrina Lacordaire, 1848		
*Anomoea rufifrons mutabilis* (Lacordaire, 1848)	Site 3 [3]	Jul (2)
	Site 4 [1]	May (1)
	Site 7 [3, 8]	Jun (7)
*Smaragdina agilis* (Lacordaire, 1848)	Site 4 [4]	Jun (1)
Subtribe Megalostomina Chapuis, 1874		
*Coscinoptera aeneipennis* (LeConte, 1858)	Site 2 [3, 7]	Jun (2)
*Coleorozena scapularis scapularis* (Lacordaire, 1848)	Site 1 [4]	Apr (1)
	Site 2 [3]	Apr (1)
*Coscinoptera tamaulipasi* Medvedev, 2012	Site [5, 9, 10, 11, 12]	Mar (4), Apr (1), May (2), Jul (1), Aug (1)
	Site 7 [2, 12]	Mar (2), May (1)
*Megalostomis dimidiata* Lacordaire, 1848	Site 1 [10]	Mar (1)
Subtribe Babiina Chapuis, 1874		
*Babia tetraspilota texana* Schaeffer, 1933	Site 1 [12]	Sep (1), Oct (1)
Tribe Fulcidacini Jakobson, 1924		
*Diplacaspis prosternalis* (Schaeffer, 1906)	Site 1 [2, 4, 9, 10]	Mar (2), Jul (2)
	Site 7 [2, 6, 8]	Mar (1), Jul (1), Aug (1)
